# Crystal structures of phosphine-supported (η^5^-cyclo­penta­dien­yl)molybdenum(II) propionyl complexes

**DOI:** 10.1107/S2056989021008008

**Published:** 2021-08-10

**Authors:** Matthew T. Whited, Margaret A. Ball, Alison Block, Benjamin A. Brewster, LouLou Ferrer, Helen J. Jin-Lee, Colby J. King, Jamie D. North, Inger L. Shelton, David G. Wilson

**Affiliations:** a Carleton College, 1 N College St, Northfield, MN 55057, USA

**Keywords:** crystal structure, phosphine, propion­yl, piano-stool complex

## Abstract

Three cyclo­penta­dienylmolybdenum(II) propionyl complexes featuring tri­aryl­phosphine ligands with different *para* substituents, namely, dicarbon­yl(η^5^-cyclo­penta­dien­yl)propion­yl(tri­phenyl­phosphane-κ*P*)molybdenum(II), [Mo(C_5_H_5_)(C_3_H_5_O)(C_18_H_15_P)(CO)_2_], (**1**), dicarbon­yl(η^5^-cyclo­penta­dien­yl)propion­yl[tris­(4-fluoro­phen­yl)phosphane-κ*P*]molybdenum(II), [Mo(C_5_H_5_)(C_3_H_5_O)(C_18_H_12_F_3_P)(CO)_2_], (**2**), and dicarbon­yl(η^5^-cyclo­penta­dien­yl)propion­yl[tris­(4-meth­oxy­phen­yl)phosphane-κ*P*]molybdenum(II) dichloromethane solvate, [Mo(C_5_H_5_)(C_3_H_5_O)(C_21_H_21_O_3_P)(CO)_2_]·CH_2_Cl_2_, (**3**), have been prepared from the corresponding ethyl complexes *via* phosphine-induced migratory insertion. These complexes exhibit four-legged piano-stool geom­etries with mol­ecular structures quite similar to each other and to related acetyl complexes. The extended structures of the three complexes differ somewhat, with the *para* substituent of the tri­aryl­phosphine of (**2**) (fluoro) or (**3**) (meth­oxy) engaging in non-classical C—H⋯F or C—H⋯O hydrogen-bonding inter­actions. The structure of (**3**) exhibits modest disorder in the position of one Cl atom of the di­chloro­methane solvent, which was modeled with two sites showing approximately equivalent occupancies [0.532 (15) and 0.478 (15)].

## Abstract 2

Solid-state structures are presented for three propionyl complexes of Mo^II^ featuring piano-stool geometries and supported by tri­aryl­phosphine ligands, showing the effects of *para* substitution on supra­molecular structure and allowing comparison to the large class of previously reported acetyl complexes.

## Chemical context   

Cyclo­penta­dienylmolybdenum(II) complexes featuring carbonyl ligands commonly adopt ‘four-legged piano-stool’ geometries (Barnett & Slocum, 1972 ▸; Kubacek *et al.*, 1982 ▸), and those featuring alkyl co-ligands readily undergo migratory insertion to afford Mo^II^ acyl complexes upon exposure to phosphines (Barnett & Treichel, 1967 ▸; Butler *et al.*, 1967 ▸). The effect of changing phosphine substituents on this reaction is well established, with bulkier phosphines enhancing the rates of subsequent deinsertion from the acyl complexes, a net deca­rbonylation (Barnett, 1969 ▸; Barnett & Pollmann, 1974 ▸). Most complexes of the type Mo(C_5_H_5_)(CO)_2_(P*R*_3_)(CO*R*) feature acetyl ligands, though there are limited examples of other acyl complexes that have been structurally characterized (Michelini-Rodriguez *et al.*, 1993 ▸; Murshid *et al.*, 2016 ▸).

We have previously reported synthetic details and solid-state structures for a number of Mo^II^ acetyl complexes of the type described above (Whited & Hofmeister, 2014 ▸; Whited *et al.*, 2012 ▸, 2014 ▸), examining the effect of changing phosphine substituents on local and supra­molecular features. Consistent with reports on deca­rbonylation reactivity, we have found that the primary impact on mol­ecular structure is observed in the Mo—P bond lengths, with some changes in P—Mo—C bond angles as a result of sterics. Use of tri(2-fur­yl)phosphine, which features heteroatoms as potential hydrogen-bond acceptors, leads to an unusual structure with the acetyl oriented down, away from the cyclo­penta­dienyl ring rather than up toward it as observed in other cases (Whited *et al.*, 2013 ▸), and a similar effect was observed by incorporation of a Lewis-acidic manganese unit to inter­act with the acetyl ligand (Adatia *et al.*, 1986 ▸). Recent use of other potentially hydrogen-bonding phosphine ligands did not lead to the same solid-state effect (Anstey *et al.*, 2020 ▸).
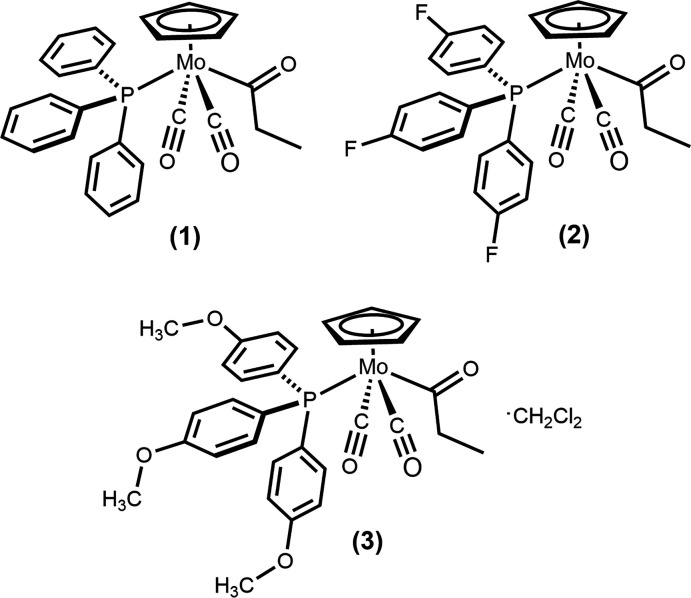


We were inter­ested in extending earlier studies to higher-order alkyl groups at molybdenum, and in this report we describe the synthesis and solid-state structures of related Mo^II^ propionyl complexes derived from an ethyl precursor and supported by tri­aryl­phosphine ligands differing in their *para* substitution (–H, –F, and –OCH_3_). Although substitution of phosphine aryl groups with electron-withdrawing or -donating groups minimally affects local structure, the supra­molecular organization is substanti­ally affected by non-classical hydrogen-bonding to the fluoro and meth­oxy groups in (**2**) and (**3**), respectively.

## Structural commentary   

**Figure 1:**
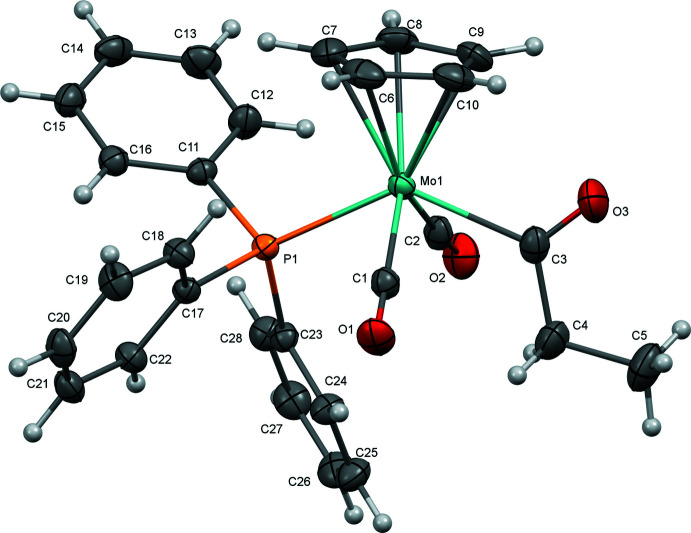
Mol­ecular structure of (**1**) with ellipsoids at 50% probability.

The mol­ecular structures of (**1**), (**2**), and (**3**) are illustrated in Figs. 1 ▸–3 ▸ ▸. All complexes exhibit an overall structure common for CpMo acetyl complexes of this type, with *trans*-disposed carbonyl ligands. As previously observed for most related acetyl complexes, the acyl C=O points up toward the Cp ring. In the case of (**1**), this phenomenon could be rationalized by presence of short C4—H4*A*⋯O1 (2.672 Å) and C4—H4*B*⋯O2 (2.639 Å) contacts involving the carbonyl ligands that are enabled when the acyl points up. However, the variation of the Mo1—C3—C4—C5 torsion angle across the series [175.31 (12)° for (**1**), 172.61 (18)° for (**2**), and 137.17 (10)° for (**3**)] argues against the general importance of such an inter­action.

Selected geometric parameters for (**1**), (**2**), and (**3**) are presented in Tables 1 ▸–3 ▸ ▸. Complex (**2**) crystallized with two nearly equivalent mol­ecules in the asymmetric unit, so geometric parameters are presented for both. In general, the three complexes are nearly identical, as might be expected based on the dominant role of sterics in determining structure and the fact that the steric profiles of the three tri­aryl­phosphine ligands are identical. The Mo—P bond length in (**2**) [2.4692 (4) Å (avg)] is slightly shorter than in (**1**) or (**3**) [2.4816 (4) and 2.4745 (3) Å, respectively], which may be related to stronger π-backbonding to the tris­(4-fluoro­phen­yl)phosphine ligand. Stronger backbonding is supported by the observation by infrared spectroscopy of slightly higher-energy carbonyl stretching vibrations for (**2**) [ν(CO)_avg_ = 1897 cm^−1^] compared with (**1**) and (**3**) [ν(CO)_avg_ = 1893 cm^−1^ for (**1**), 1890 cm^−1^ for (**3**)]. Geometric parameters for all complexes are quite similar to those for the related tri­phenyl­phosphine-supported CpMo acetyl complex (Churchill & Fennessey, 1968 ▸).

## Supra­molecular features   

**Figure 4:**
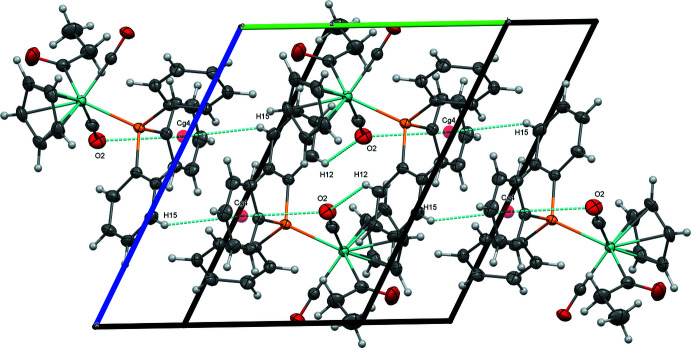
Chains of (**1**) along [010], viewed along [3
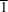
0].

**Figure 5:**
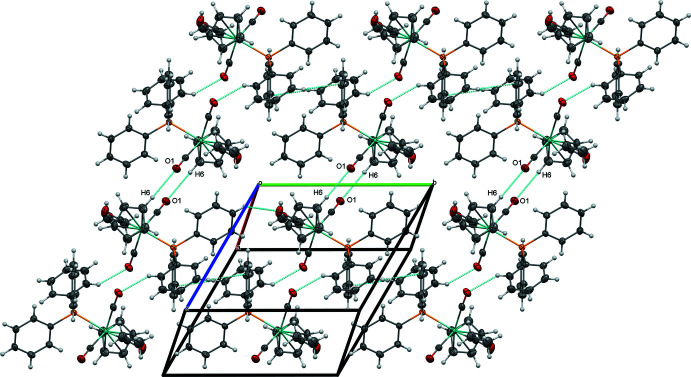
Sheets of (**1**) formed by C—H⋯O inter­actions, viewed perpendicular to (10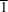).

In spite of the similarities among (**1**), (**2**), and (**3**) in their mol­ecular structures, the *para* substituent of the tri­aryl­phosphine ligand [H for (**1**), F for (**2**), OCH_3_ for (**3**)] plays an important role in determining the extended structure. The extended structure of (**1**) is dominated by non-classical C—H⋯O inter­actions involving its carbonyl ligands. A short C—H⋯O inter­action between O2 and H12 of a phenyl ring (2.36 Å) joins mol­ecules of (**1**) into centrosymmetrical dimers that are organized into chains along [010] by inter­molecular C15—H15⋯*Cg*4 (2.952 Å, where *Cg*4 represents the centroid of the C23–C28 ring) and intra­molecular O2⋯*Cg*4 (3.295 Å) inter­actions (Fig. 4 ▸, Table 4 ▸). These chains are linked into sheets parallel to (10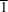) through another set of non-classical C—H⋯O inter­actions (2.60 Å) between O1 of the other carbonyl ligand and H6 from a cyclo­penta­dienyl ligand (Fig. 5 ▸).

**Figure 6:**
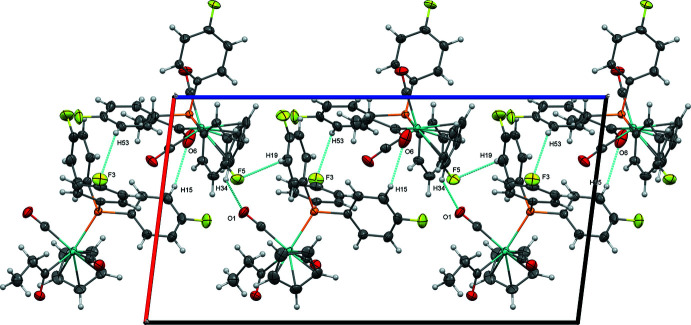
Chains along [001] of the two nearly identical mol­ecules of (**2**) in the asymmetric unit, with their C—H⋯O and C—H⋯F inter­actions, viewed along [010].

**Figure 7:**
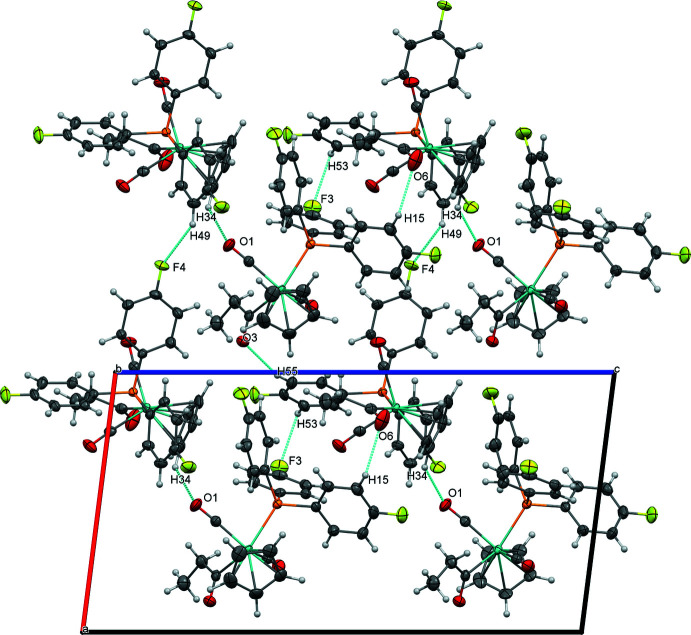
Sheets of (**2**) parallel to (010), viewed perpendicular to (010).

The tris­(4-fluoro­phen­yl)phosphine-supported derivative (**2**) features two nearly equivalent mol­ecules in the asymmetric unit exhibiting a non-classical C—H⋯O inter­action between O6 of a propionyl ligand and H15 from a phenyl ring (2.59 Å) and a C—H⋯F close contact between F3 and H53 (2.60 Å). These pairs of mol­ecules are joined into chains along [001] by C34—H34⋯O1 hydrogen bonding (2.38 Å, Fig. 6 ▸, Table 5 ▸)). The mol­ecules are further organized parallel to (010) by C—H⋯F close contacts between F4 and H49 (2.55 Å) and C—H⋯O inter­actions between O3 and H55 (2.53 Å) (Fig. 7 ▸), then further joined along [010] by C—F⋯π inter­actions (F5⋯*Cg*4 = 3.17 Å, where *Cg*4 represents the centroid of the C23–C38 ring).

**Figure 8:**
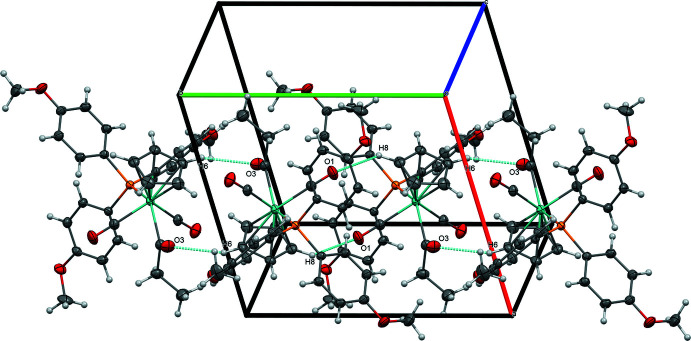
Chains of (**3**) along [010], viewed perpendicular to (001).

**Figure 9:**
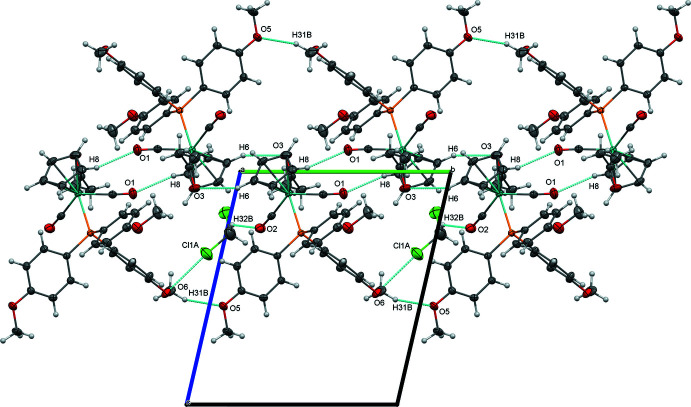
Network of complex (**3**) formed by inter­actions featuring meth­oxy groups and di­chloro­methane solvent, viewed along [100].

Like complex (**1**), complex (**3**) is joined into centrosymmetrical dimers by a C—H⋯O inter­action involving a carbonyl ligand (C8—H8⋯O1, Table 6 ▸), and these are linked into chains along [110] through an additional C—H⋯O inter­action between the propionyl oxygen and a cyclo­penta­dienyl ligand (Fig. 8 ▸). Additional C31—H31*B*⋯O5 inter­actions along [110], involving a meth­oxy group from the phosphine ligand, join the mol­ecules into a network parallel to (001). This further set of inter­actions involving meth­oxy groups, as well as important close contacts involving the di­chloro­methane solvent, are depicted in Fig. 9 ▸.

## Database survey   

The current version of the Cambridge Structural Database (Version 5.41, updated August 2020; Groom *et al.*, 2016 ▸) has fourteen entries corresponding to molybdenum acyl complexes of the general form Mo(C_5_H_5_)(CO)_2_(P*R*_3_)(CO*R*). The *trans*-dicarbonyl structure, as observed for (**1**)–(**3**), is preferred except in cases where the phosphine and acyl ligands are covalently linked, forcing them to be *cis* (Adams *et al.*, 1991 ▸; Mercier *et al.*, 1993 ▸; Yan *et al.*, 2009 ▸).

## Synthesis and crystallization   

**CpMo(CO)_3_(CH_2_CH_3_)**. This compound was prepared by modification of the method used of Gladysz *et al.* (1979 ▸), as previously reported by Whited & Hofmeister (2014 ▸) and Anstey *et al.* (2020 ▸). In a 20 ml scintillation vial equipped with a flea-sized stir bar, [CpMo(CO)_3_]_2_ (0.1908 g, 0.39 mmol) was dissolved in THF (10 ml). Sodium tri­ethyl­borohydride (0.87 mL of 1.0 *M* solution in THF, 0.87 mmol) was added dropwise by syringe with vigorous stirring, leading to an immediate color change from purple to green–yellow with evolution of H_2_ gas. The reaction was allowed to proceed with stirring for 20 min, and an excess of iodo­ethane (0.098 ml, 1.2 mmol) was added dropwise with stirring and the reaction was allowed to proceed for 6 h. Volatiles were removed *in vacuo* to afford a yellow–brown film that was stored at 238 K for 1 week. The solid was extracted with pentane (4 × 10 ml) and filtered through a 1 cm pad of activated alumina to afford a yellow solution, and removal of solvent *in vacuo* afforded **CpMo(CO)_3_(CH_2_CH_3_)** as a pure yellow powder (0.131 g, 61%). ^1^H NMR (400 MHz, CDCl_3_): δ 5.28 (*s*, 5H, Cp ring), 1.72 (*q*, ^3^ *J*_HH_ = 7.4 Hz, 2H, –C*H*_2_CH_3_), 1.45 (*t*, ^3^ *J*_HH_ = 7.4 Hz, 3H, –CH_2_C*H*_3_). ^13^C{^1^H} NMR (101 MHz, CDCl_3_): δ 239.9 (Mo—*C*O), 227.8 (Mo—*C*O), 93.0 (Cp ring), 20.4 (Mo—CH_2_ *C*H_3_) −3.7 (Mo—*C*H_2_CH_3_). IR (CH_2_Cl_2_, NaCl, cm^−1^) ν(CO): 2015, 1921 (split).

**CpMo(CO)_2_(PPh_3_)(COCH_2_CH_3_) (1)**. In an inert-atmos­phere glove box, CpMo(CO)_3_(CH_2_CH_3_) (0.0803 g, 0.293 mmol) and tri­phenyl­phosphine (0.115 g, 0.440 mmol, 1.5 equiv) were dissolved in aceto­nitrile (5 ml) in a 20 ml scintillation vial equipped with a flea-sized stir bar. The mixture was stirred for 1 week, during which time a bright-yellow precip­itate formed. The yellow solid was isolated by filtration and washed with pentane (2 × 5 ml), then dried *in vacuo* to afford pure **1**. Yellow crystals of **1** suitable for X-ray diffraction were obtained from a concentrated di­chloro­methane solution by vapor cross diffusion with pentane at 238 K. ^1^H NMR (400 MHz, CDCl_3_): δ 7.50–7.30 (*m*, 15H, PPh_3_), 5.00 (*d*, *J* = 1.2 Hz, 5H, C_5_ *H_5_*), 3.03 (*q*, ^3^ *J*_HH_ = 7.2 Hz, 2H, C(O)C*H*_2_CH_3_), 0.90 (*t*, ^3^ *J*_HH_ = 7.2 Hz, 3H, C(O)CH_2_C*H*_3_). ^13^C{^1^H} NMR (101 MHz, CDCl_3_): δ 267.7 (*d*, ^2^ *J*_CP_ = 11 Hz, Mo—*C*OEt), 238.8 (*d*, ^2^ *J*_CP_ = 24 Hz, Mo—*C*O), 135.7 (*d*, ^1^ *J*_CP_ = 44 Hz, *ipso*-C of PPh_3_), 133.2 (*d*, ^2^ *J*_CP_ = 11 Hz, *ortho*-C of PPh_3_), 130.5 (*d*, ^4^ *J*_CP_ = 2 Hz, *para*-C of PPh_3_), 128.6 (*d*, ^3^ *J*_CP_ = 11 Hz, *meta*-C of PPh_3_), 96.7 (Cp ring), 58.1 (Mo—CO*C*H_2_CH_3_), 10.1 (Mo—COCH_2_ *C*H_3_). ^31^P{^1^H} NMR (162 MHz, CDCl_3_): δ 68.4 (*s*). IR (CH_2_Cl_2_, NaCl, cm^−1^) ν(CO): 1935, 1851, 1614 (acet­yl).

**CpMo(CO)_2_(P(4-FPh)_3_)(COCH_2_CH_3_) (2)**. In an inert-atmosphere glove box, CpMo(CO)_3_(CH_2_CH_3_) (0.0997 g, 0.36 mmol) and tris­(4-fluoro­phen­yl)phosphine (0.17 g, 0.55 mmol, 1.5 equiv) were dissolved in aceto­nitrile (5 ml) in a 20 ml scintillation vial equipped with a flea-sized stir bar. The mixture was stirred for 1 week, causing a color change to orange, but without formation of any precipitate. Solvent was removed *in vacuo*, causing precipitation of a yellow solid that was isolated by filtration and washed with pentane (2 × 3 ml) to afford the desired product **2** (0.12 g, 56%). Yellow crystals of **2** suitable for X-ray diffraction were obtained from a concentrated di­chloro­methane solution by vapor cross diffusion with pentane at 238 K. ^1^H NMR (400 MHz, CDCl_3_): δ 7.41–7.30 (*br m*, 6H, *ortho*-C–*H* of phosphine), 7.14 (*td*, ^3^ *J*_HH_ ≃ ^3^ *J*_HF_ = 8.6 Hz, ^4^ *J*_HP_ = 1.5 Hz, 6H, *meta*-C—*H* of phosphine), 4.90 (*d*, *J* = 1.2 Hz, 5H, C_5_ *H_5_*), 2.99 (*q*, ^3^ *J*_HH_ = 7.2 Hz, 2H, C(O)C*H*_2_CH_3_), 0.90 (*t*, ^3^ *J*_HH_ = 7.2 Hz, 3H, C(O)CH_2_C*H*_3_). ^13^C{^1^H} NMR (101 MHz, CDCl_3_): δ 265.4 (*d*, ^2^ *J*_CP_ = 11 Hz, Mo—*C*OEt), 238.2 (*d*, ^2^ *J*_CP_ = 24 Hz, Mo—*C*O), 164.0 (*dd*, ^1^ *J*_CF_ = 253 Hz, ^4^ *J*_CP_ = 2 Hz, *C*—F of phosphine), 135.0 (*dd*, ^2^ *J*_CP_ = 13 Hz, ^3^ *J*_CF_ = 8 Hz, *ortho*-C of phosphine), 131.3 (*dd*, ^1^ *J*_CP_ = 46 Hz, ^4^ *J*_CF_ = 4 Hz, *ipso*-C of phosphine), 116.0 (*dd*, ^2^ *J*_CF_ = 21 Hz, ^3^ *J*_CP_ = 11 Hz, *meta*-C of phosphine), 96.5 (Cp ring), 58.2 (Mo—CO*C*H_2_CH_3_), 10.9 (Mo—COCH_2_ *C*H_3_). ^31^P{^1^H} NMR (162 MHz, CDCl_3_): δ 68.5 (*s*). IR (CH_2_Cl_2_, NaCl, cm^−1^) ν(CO): 1938, 1856, 1620 (acet­yl).

**CpMo(CO)_2_(P(4-MeOPh)_3_)(COCH_2_CH_3_) (3)**. In an inert-atmosphere glove box, CpMo(CO)_3_(CH_2_CH_3_) (0.113 g, 0.41 mmol) and tris­(4-meth­oxy­phen­yl)phosphine (0.218 g, 0.61 mmol, 1.5 equiv) were dissolved in aceto­nitrile (5 ml) in a 20 nml scintillation vial equipped with a flea-sized stir bar. The mixture was stirred for 1 week, causing precipitation of **3** as a pure yellow powder that was isolated by filtration. Crystals of **3** suitable for X-ray diffraction were obtained from a concentrated di­chloro­methane solution by vapor cross diffusion with pentane at 238 K. ^1^H NMR (400 MHz, CDCl_3_): δ 7.37–7.23 (*br m*, 6H, *ortho*-C–*H* of phosphine), 7.14 (*dd*, ^3^ *J*_HH_ = 8.8 Hz, ^4^ *J*_HP_ = 1.7 Hz, *meta*-C—*H* of phosphine), 4.99 (*d*, *J* = 1.2 Hz, 5H, C_5_ *H_5_*), 3.03 (*q*, ^3^ *J*_HH_ = 7.2 Hz, 2H, C(O)C*H*_2_CH_3_), 0.89 (*t*, ^3^ *J*_HH_ = 7.2 Hz, 3H, C(O)CH_2_C*H*_3_). ^13^C{^1^H} NMR (101 MHz, CDCl_3_): δ 268.6 (*d*, ^2^ *J*_CP_ = 11 Hz, Mo–*C*OEt), 239.2 (*d*, ^2^ *J*_CP_ = 24 Hz, Mo—*C*O), 161.1 (*C*—OCH_3_ of phosphine), 134.6 (*d*, ^2^ *J*_CP_ = 12 Hz, *ortho*-C of phosphine), 127.4 (*d*, ^1^ *J*_CP_ = 50 Hz, *ipso*-C of phosphine), 114.0 (*d*, ^3^ *J*_CP_ = 11 Hz, *meta*-C of phosphine), 96.6 (Cp ring), 58.0 (Mo—CO*C*H_2_CH_3_), 10.1 (Mo—COCH_2_ *C*H_3_). ^31^P{^1^H} NMR (162 MHz, CDCl_3_): δ 62.3 (*s*). IR (CH_2_Cl_2_, NaCl, cm^−1^) ν(CO): 1933, 1847, 1605 (acet­yl).

## Refinement   

Crystal data, data collection and structure refinement details are summarized in Table 7 ▸. H atoms were placed in calculated positions and refined in the riding-model approximation with distances of C—H = 0.95, 0.98, 0.99, and 1.00 Å for the phenyl, methyl, methyl­ene, and cyclo­penta­dienyl groups, respectively, and with *U*_iso_(H) = *k*×*U*_eq_(C), *k* = 1.2 for cyclo­penta­dienyl, phenyl, and methyl­ene groups and 1.5 for methyl groups. Methyl group H atoms were allowed to rotate in order to find the best rotameric conformation.

A small number of intense low-angle reflections [three for (**1**); seven for (**2**); five for (**3**)] are missing from these high-quality data sets due to the arrangement of the instrument with a conservatively sized beam stop. The large number of reflections in the data sets (and the Fourier-transform relationship of intensities to atoms) ensures that no particular bias has been introduced.

The structure of (**3**) exhibits modest disorder in the position of Cl1 of the di­chloro­methane solvent, which was modeled with two sites showing approximately equivalent occupancies [0.532 (15) for Cl1*A*, 0.468 (15) for Cl1*B*].

## Supplementary Material

Crystal structure: contains datablock(s) global, 1, 2, 3. DOI: 10.1107/S2056989021008008/jq2008sup1.cif

Structure factors: contains datablock(s) 1. DOI: 10.1107/S2056989021008008/jq20081sup2.hkl

Supporting information file. DOI: 10.1107/S2056989021008008/jq20081sup5.cdx

Structure factors: contains datablock(s) 2. DOI: 10.1107/S2056989021008008/jq20082sup3.hkl

Supporting information file. DOI: 10.1107/S2056989021008008/jq20082sup6.cdx

Structure factors: contains datablock(s) 3. DOI: 10.1107/S2056989021008008/jq20083sup4.hkl

Supporting information file. DOI: 10.1107/S2056989021008008/jq20083sup7.cdx

CCDC references: 2101246, 2101245, 2101244

Additional supporting information:  crystallographic information; 3D view; checkCIF report

## Figures and Tables

**Figure 2:**
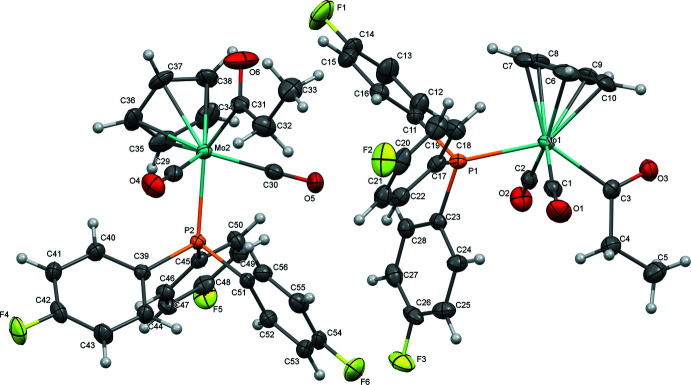
Mol­ecular structure of (**2**) with ellipsoids at 50% probability.

**Figure 3:**
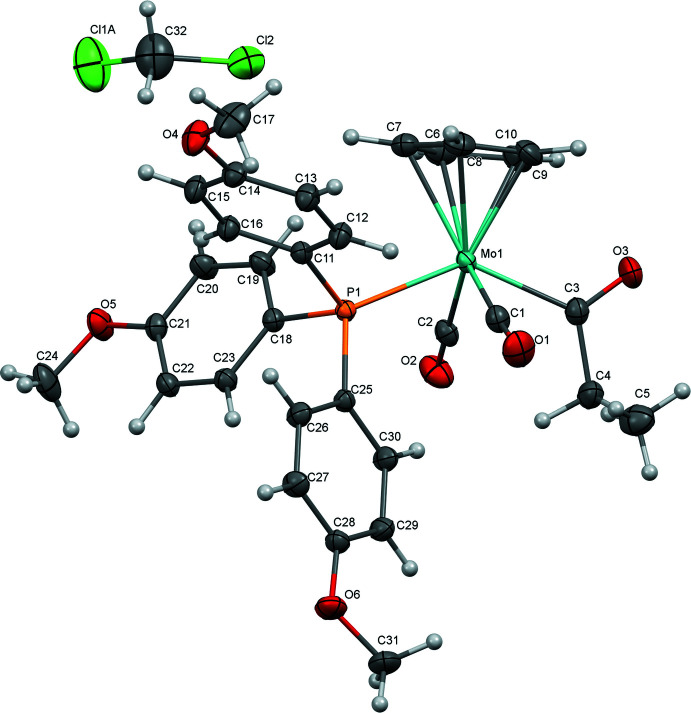
Mol­ecular structure of (**3**) with ellipsoids at 50% probability.

**Table 1:** Selected geometric parameters (Å, °) for (**1**)

Mo1—P1	2.4816 (4)	Mo1—C2	1.9662 (15)
Mo1—C1	1.9640 (13)	Mo1—C3	2.2794 (14)
			
C1—Mo1—C2	107.83 (6)	C1—Mo1—P1	79.34 (4)
C1—Mo1—C3	74.37 (5)	C2—Mo1—P1	78.28 (4)
C2—Mo1—C3	72.47 (5)	C3—Mo1—P1	131.76 (4)
			
Mo1—C3—C4—C5	175.31 (12)		

**Table 2:** Selected geometric parameters (Å, °) for (**2**)

Mo1—P1	2.4730 (6)	Mo2—C29	1.962 (2)
Mo1—C1	1.966 (2)	Mo2—C30	1.964 (3)
Mo1—C2	1.965 (2)	Mo2—C31	2.286 (2)
Mo1—C3	2.265 (2)	Mo2—P2	2.4654 (6)
			
C1—Mo1—C2	109.77 (10)	C29—Mo2—C30	107.12 (10)
C1—Mo1—C3	77.34 (9)	C29—Mo2—C31	74.64 (9)
C2—Mo1—C3	70.93 (9)	C30—Mo2—C31	73.89 (9)
C1—Mo1—P1	80.12 (7)	C29—Mo2—P2	78.59 (7)
C2—Mo1—P1	79.50 (7)	C30—Mo2—P2	78.48 (7)
C3—Mo1—P1	133.55 (6)	C31—Mo2—P2	133.28 (6)
			
Mo1—C3—C4—C5	172.61 (18)	Mo2—C31—C32—C33	173.63 (19)

**Table 3:** Selected geometric parameters (Å, °) for (**3**)

Mo1—P1	2.4745 (3)	Mo1—C2	1.9658 (12)
Mo1—C1	1.9675 (12)	Mo1—C3	2.2564 (11)
			
C1—Mo1—C2	106.36 (5)	C1—Mo1—P1	79.97 (3)
C1—Mo1—C3	72.49 (4)	C2—Mo1—P1	79.79 (3)
C2—Mo1—C3	74.79 (4)	C3—Mo1—P1	134.90 (3)
			
Mo1—C3—C4—C5	137.17 (10)		

**Table 4:** Hydrogen-bond geometry (Å, °) for (**1**)

*D*—H⋯*A*	*D*—H	H⋯*A*	*D*⋯*A*	*D*—H⋯*A*
C12—H12⋯O2^i^	0.95	2.36	3.1188 (18)	137
C6—H6⋯O1^ii^	1.00	2.60	3.545 (2)	158

Symmetry codes: (i) -x+1, -y+1, -z+1; (ii) -x, -y+1, -z.

**Table 5:** Hydrogen-bond geometry (Å, °) for (**2**)

*D*—H⋯*A*	*D*—H	H⋯*A*	*D*⋯*A*	*D*—H⋯*A*
C15—H15⋯O6	0.95	2.59	3.371 (4)	139
C49—H49⋯F4^i^	0.95	2.55	3.344 (3)	141
C55—H55⋯O3^ii^	0.95	2.53	3.450 (3)	164
C34—H34⋯O1^iii^	1.00	2.38	3.237 (3)	143

Symmetry codes: (i) x+1, y, z; (ii) x-1, y, z; (iii) x, -y+{\script{1\over 2}}, z+{\script{1\over 2}}.

**Table 6:** Hydrogen-bond geometry (Å, °) for (**3**)

*D*—H⋯*A*	*D*—H	H⋯*A*	*D*⋯*A*	*D*—H⋯*A*
C31—H31*B*⋯O5^i^	0.98	2.58	3.4880 (16)	155
C6—H6⋯O3^ii^	1.00	2.57	3.4555 (16)	148
C8—H8⋯O1^iii^	1.00	2.45	3.2714 (16)	139
C32—H32*B*⋯O2^iv^	0.99	2.63	3.418 (2)	137

Symmetry codes: (i) x+1, y+1, z; (ii) -x+1, -y, -z; (iii) -x+1, -y+1, -z; (iv) x-1, y, z.

**Table 7:** Experimental details

	(**1**)	(**2**)	(**3**)
Crystal data			
Chemical formula	[Mo(C_5_H_5_)(C_3_H_5_O)(C_18_H_15_P)(CO)_2_]	[Mo(C_5_H_5_)(C_3_H_5_O)(C_18_H_12_F_3_P)(CO)_2_]	[Mo(C_5_H_5_)(C_3_H_5_O)(C_21_H_21_O_3_P)(CO)_2_]·CH_2_Cl_2_
*M*_r_	536.39	590.36	711.39
Crystal system, space group	Triclinic, *P*\overline{1}	Monoclinic, *P*2_1_/*c*	Triclinic, *P*\overline{1}
Temperature (K)	170	170	170
*a*, *b*, *c* (Å)	9.1719 (5), 11.7493 (7), 12.6049 (7)	11.7991 (4), 18.6907 (8), 22.4744 (8)	10.5308 (6), 12.1305 (7), 13.6154 (8)
α, β, γ (°)	113.083 (2), 99.148 (2), 99.380 (2)	90, 97.256 (2), 90	97.660 (2), 104.759 (2), 107.081 (2)
*V* (Å^3^)	1195.14 (12)	4916.7 (3)	1566.43 (16)
*Z*	2	8	2
Radiation type	Mo *K*α	Mo *K*α	Mo *K*α
μ (mm^−1^)	0.64	0.65	0.68
Crystal size (mm)	0.16 × 0.16 × 0.06	0.05 × 0.05 × 0.05	0.23 × 0.21 × 0.12

Data collection			
Diffractometer	Bruker D8 QUEST ECO	Bruker D8 QUEST ECO	Bruker D8 QUEST ECO
Absorption correction	Multi-scan (Krause *et al.*, 2015 ▸)	Multi-scan (Krause *et al.*, 2015 ▸)	Multi-scan (Krause *et al.*, 2015 ▸)
*T*_min_, *T*_max_	0.89, 0.96	0.86, 0.97	0.84, 0.92
No. of measured, independent and observed [*I* > 2σ(*I*)] reflections	49481, 5939, 5674	73758, 10047, 8547	81820, 9578, 9053
*R*_int_	0.025	0.042	0.029
(sin θ/λ)_max_ (Å^−1^)	0.667	0.625	0.714

Refinement			
*R*[*F*^2^ > 2σ(*F* ^2^)], *wR*(*F* ^2^), *S*	0.019, 0.051, 1.07	0.029, 0.063, 1.07	0.020, 0.054, 1.06
No. of reflections	5939	10047	9578
No. of parameters	299	651	393
H-atom treatment	H-atom parameters constrained	H-atom parameters constrained	H-atom parameters constrained
Δρ_max_, Δρ_min_ (e Å^−3^)	0.34, −0.40	0.37, −0.39	0.40, −0.48

Computer programs: *BIS* and *SAINT* (Bruker, 2019 ▸), *SHELXT2018/2* (Sheldrick, 2015*a* ▸), *SHELXL2018/3* (Sheldrick, 2015*b* ▸), *Mercury* (Macrae *et al.*, 2020 ▸), and *publCIF* (Westrip, 2010 ▸).

## supplementary crystallographic information

### Dicarbonyl(η5-cyclopentadienyl)propionyl(triphenylphosphane-κP)molybdenum(II) (1) Crystal data

**Table:**

[Mo(C_5_H_5_)(C_3_H_5_O)(C_18_H_15_P)(CO)_2_]	*Z* = 2
*M**_r_* = 536.39	*F*(000) = 548
Triclinic, *P*1	*D*_x_ = 1.491 Mg m^−^^3^
*a* = 9.1719 (5) Å	Mo *K*α radiation, λ = 0.71073 Å
*b* = 11.7493 (7) Å	Cell parameters from 9085 reflections
*c* = 12.6049 (7) Å	θ = 2.6–33.1°
α = 113.083 (2)°	µ = 0.64 mm^−^^1^
β = 99.148 (2)°	*T* = 170 K
γ = 99.380 (2)°	Prism, yellow
*V* = 1195.14 (12) Å^3^	0.16 × 0.16 × 0.06 mm

### Dicarbonyl(η5-cyclopentadienyl)propionyl(triphenylphosphane-κP)molybdenum(II) (1) Data collection

**Table:**

Bruker D8 QUEST ECO diffractometer	5939 independent reflections
Radiation source: sealed tube, Siemens KFFMO2K-90C	5674 reflections with *I* > 2σ(*I*)
Curved Graphite monochromator	*R*_int_ = 0.025
Detector resolution: 7.3910 pixels mm^-1^	θ_max_ = 28.3°, θ_min_ = 2.3°
φ and ω scans	*h* = −12→12
Absorption correction: multi-scan (Krause *et al.*, 2015)	*k* = −15→15
*T*_min_ = 0.89, *T*_max_ = 0.96	*l* = −16→16
49481 measured reflections	

### Dicarbonyl(η5-cyclopentadienyl)propionyl(triphenylphosphane-κP)molybdenum(II) (1) Refinement

**Table:**

Refinement on *F*^2^	Primary atom site location: dual
Least-squares matrix: full	Secondary atom site location: difference Fourier map
*R*[*F*^2^ > 2σ(*F*^2^)] = 0.019	Hydrogen site location: inferred from neighbouring sites
*wR*(*F*^2^) = 0.051	H-atom parameters constrained
*S* = 1.07	*w* = 1/[σ^2^(*F*_o_^2^) + (0.022*P*)^2^ + 0.6108*P*] where *P* = (*F*_o_^2^ + 2*F*_c_^2^)/3
5939 reflections	(Δ/σ)_max_ = 0.001
299 parameters	Δρ_max_ = 0.34 e Å^−^^3^
0 restraints	Δρ_min_ = −0.40 e Å^−^^3^

### Dicarbonyl(η5-cyclopentadienyl)propionyl(triphenylphosphane-κP)molybdenum(II) (1) Special details

**Table:**

Geometry. All esds (except the esd in the dihedral angle between two l.s. planes) are estimated using the full covariance matrix. The cell esds are taken into account individually in the estimation of esds in distances, angles and torsion angles; correlations between esds in cell parameters are only used when they are defined by crystal symmetry. An approximate (isotropic) treatment of cell esds is used for estimating esds involving l.s. planes.

### Dicarbonyl(η5-cyclopentadienyl)propionyl(triphenylphosphane-κP)molybdenum(II) (1) Fractional atomic coordinates and isotropic or equivalent isotropic displacement parameters (Å^2^)

**Table:**

	*x*	*y*	*z*	*U*_iso_*/*U*_eq_	
Mo1	0.21851 (2)	0.45813 (2)	0.23960 (2)	0.01978 (4)	
P1	0.32246 (3)	0.69322 (3)	0.33370 (3)	0.01786 (6)	
O1	0.22653 (14)	0.50413 (11)	0.01296 (9)	0.0343 (2)	
O2	0.54687 (13)	0.48265 (11)	0.37404 (11)	0.0391 (3)	
O3	0.23712 (16)	0.18616 (11)	0.08762 (12)	0.0459 (3)	
C1	0.22950 (15)	0.48927 (12)	0.09861 (11)	0.0233 (2)	
C2	0.42735 (17)	0.47555 (13)	0.32222 (12)	0.0267 (3)	
C3	0.30515 (18)	0.29556 (13)	0.11888 (12)	0.0288 (3)	
C4	0.4478 (2)	0.32080 (16)	0.07449 (15)	0.0390 (4)	
H4A	0.428632	0.366718	0.024555	0.047*	
H4B	0.533049	0.37787	0.144096	0.047*	
C5	0.4970 (2)	0.20261 (19)	0.00328 (17)	0.0504 (5)	
H5A	0.420288	0.151328	−0.072025	0.076*	
H5B	0.507534	0.15204	0.048824	0.076*	
H5C	0.595154	0.227972	−0.013045	0.076*	
C6	−0.05044 (17)	0.42011 (17)	0.21381 (16)	0.0377 (4)	
H6	−0.121988	0.444525	0.162697	0.045*	
C7	0.01630 (18)	0.49182 (16)	0.33645 (15)	0.0356 (3)	
H7	0.00108	0.576933	0.387033	0.043*	
C8	0.09701 (19)	0.41922 (16)	0.37808 (14)	0.0348 (3)	
H8	0.146453	0.442892	0.463005	0.042*	
C9	0.08355 (19)	0.30236 (15)	0.28055 (15)	0.0354 (3)	
H9	0.118582	0.227614	0.284712	0.042*	
C10	−0.00792 (19)	0.30284 (16)	0.17850 (15)	0.0369 (3)	
H10	−0.047308	0.228557	0.098522	0.044*	
C11	0.29484 (14)	0.77386 (12)	0.48373 (11)	0.0209 (2)	
C12	0.32519 (18)	0.71890 (14)	0.56225 (12)	0.0296 (3)	
H12	0.360821	0.643324	0.537306	0.035*	
C13	0.30390 (19)	0.77344 (15)	0.67645 (13)	0.0337 (3)	
H13	0.325727	0.735596	0.729406	0.04*	
C14	0.25087 (18)	0.88295 (15)	0.71309 (13)	0.0329 (3)	
H14	0.235238	0.919963	0.790907	0.039*	
C15	0.22073 (17)	0.93831 (14)	0.63604 (13)	0.0309 (3)	
H15	0.1844	1.013525	0.661221	0.037*	
C16	0.24327 (15)	0.88454 (12)	0.52163 (12)	0.0238 (2)	
H16	0.223316	0.923696	0.46953	0.029*	
C17	0.25504 (14)	0.78737 (12)	0.25929 (11)	0.0190 (2)	
C18	0.10476 (15)	0.74751 (13)	0.19178 (12)	0.0240 (2)	
H18	0.041175	0.669342	0.180761	0.029*	
C19	0.04765 (17)	0.82174 (14)	0.14056 (13)	0.0302 (3)	
H19	−0.055523	0.795183	0.096435	0.036*	
C20	0.14075 (18)	0.93438 (14)	0.15365 (13)	0.0305 (3)	
H20	0.101594	0.984707	0.118267	0.037*	
C21	0.29082 (18)	0.97327 (13)	0.21836 (13)	0.0287 (3)	
H21	0.355199	1.049602	0.226151	0.034*	
C22	0.34774 (15)	0.90100 (12)	0.27206 (12)	0.0242 (3)	
H22	0.450298	0.929118	0.317696	0.029*	
C23	0.52909 (14)	0.73720 (12)	0.35437 (11)	0.0207 (2)	
C24	0.58606 (15)	0.69609 (13)	0.25280 (12)	0.0258 (3)	
H24	0.5177	0.656619	0.176174	0.031*	
C25	0.74111 (16)	0.71250 (15)	0.26317 (14)	0.0319 (3)	
H25	0.779018	0.68406	0.1938	0.038*	
C26	0.84183 (16)	0.77070 (15)	0.37523 (15)	0.0331 (3)	
H26	0.948349	0.781057	0.382243	0.04*	
C27	0.78743 (16)	0.81325 (14)	0.47583 (13)	0.0310 (3)	
H27	0.856515	0.853859	0.552162	0.037*	
C28	0.63068 (16)	0.79681 (13)	0.46591 (12)	0.0264 (3)	
H28	0.593438	0.826442	0.53553	0.032*	

### Dicarbonyl(η5-cyclopentadienyl)propionyl(triphenylphosphane-κP)molybdenum(II) (1) Atomic displacement parameters (Å^2^)

**Table:**

	*U*^11^	*U*^22^	*U*^33^	*U*^12^	*U*^13^	*U*^23^
Mo1	0.02476 (6)	0.01872 (6)	0.01856 (6)	0.00571 (4)	0.00643 (4)	0.01018 (4)
P1	0.01821 (14)	0.01985 (14)	0.01678 (14)	0.00582 (11)	0.00409 (11)	0.00879 (11)
O1	0.0494 (6)	0.0349 (6)	0.0221 (5)	0.0107 (5)	0.0090 (4)	0.0155 (4)
O2	0.0397 (6)	0.0413 (6)	0.0407 (6)	0.0187 (5)	0.0014 (5)	0.0220 (5)
O3	0.0611 (8)	0.0230 (5)	0.0525 (7)	0.0117 (5)	0.0214 (6)	0.0118 (5)
C1	0.0263 (6)	0.0203 (6)	0.0214 (6)	0.0053 (5)	0.0048 (5)	0.0078 (5)
C2	0.0368 (7)	0.0246 (6)	0.0237 (6)	0.0131 (5)	0.0088 (5)	0.0129 (5)
C3	0.0399 (8)	0.0254 (6)	0.0231 (6)	0.0133 (6)	0.0073 (6)	0.0105 (5)
C4	0.0481 (9)	0.0354 (8)	0.0361 (8)	0.0188 (7)	0.0199 (7)	0.0112 (7)
C5	0.0631 (12)	0.0475 (10)	0.0411 (9)	0.0299 (9)	0.0218 (9)	0.0097 (8)
C6	0.0248 (7)	0.0488 (9)	0.0495 (9)	0.0041 (6)	0.0104 (6)	0.0327 (8)
C7	0.0338 (8)	0.0335 (8)	0.0460 (9)	0.0091 (6)	0.0235 (7)	0.0183 (7)
C8	0.0409 (8)	0.0411 (8)	0.0281 (7)	0.0056 (7)	0.0165 (6)	0.0194 (6)
C9	0.0462 (9)	0.0290 (7)	0.0416 (8)	0.0071 (6)	0.0193 (7)	0.0236 (7)
C10	0.0378 (8)	0.0338 (8)	0.0336 (8)	−0.0071 (6)	0.0109 (6)	0.0142 (6)
C11	0.0198 (5)	0.0229 (6)	0.0189 (5)	0.0043 (5)	0.0048 (4)	0.0083 (5)
C12	0.0393 (8)	0.0309 (7)	0.0231 (6)	0.0137 (6)	0.0095 (6)	0.0136 (6)
C13	0.0438 (8)	0.0366 (8)	0.0220 (6)	0.0064 (6)	0.0086 (6)	0.0149 (6)
C14	0.0375 (8)	0.0317 (7)	0.0217 (6)	0.0000 (6)	0.0117 (6)	0.0052 (5)
C15	0.0332 (7)	0.0243 (6)	0.0296 (7)	0.0057 (5)	0.0120 (6)	0.0050 (5)
C16	0.0231 (6)	0.0221 (6)	0.0238 (6)	0.0034 (5)	0.0054 (5)	0.0085 (5)
C17	0.0209 (5)	0.0210 (6)	0.0176 (5)	0.0069 (4)	0.0057 (4)	0.0096 (5)
C18	0.0217 (6)	0.0248 (6)	0.0265 (6)	0.0047 (5)	0.0041 (5)	0.0130 (5)
C19	0.0272 (7)	0.0330 (7)	0.0308 (7)	0.0092 (6)	−0.0002 (5)	0.0163 (6)
C20	0.0430 (8)	0.0266 (7)	0.0270 (7)	0.0144 (6)	0.0060 (6)	0.0153 (6)
C21	0.0382 (7)	0.0215 (6)	0.0279 (7)	0.0054 (5)	0.0083 (6)	0.0127 (5)
C22	0.0244 (6)	0.0226 (6)	0.0245 (6)	0.0033 (5)	0.0041 (5)	0.0107 (5)
C23	0.0192 (5)	0.0212 (6)	0.0223 (6)	0.0064 (4)	0.0043 (5)	0.0096 (5)
C24	0.0218 (6)	0.0286 (7)	0.0229 (6)	0.0043 (5)	0.0043 (5)	0.0080 (5)
C25	0.0255 (7)	0.0342 (7)	0.0335 (7)	0.0090 (6)	0.0119 (6)	0.0097 (6)
C26	0.0187 (6)	0.0348 (7)	0.0442 (8)	0.0079 (5)	0.0050 (6)	0.0158 (7)
C27	0.0250 (6)	0.0325 (7)	0.0302 (7)	0.0037 (5)	−0.0029 (5)	0.0132 (6)
C28	0.0254 (6)	0.0281 (6)	0.0231 (6)	0.0063 (5)	0.0029 (5)	0.0096 (5)

### Dicarbonyl(η5-cyclopentadienyl)propionyl(triphenylphosphane-κP)molybdenum(II) (1) Geometric parameters (Å, º)

**Table:**

Mo1—P1	2.4816 (4)	C11—C12	1.3951 (18)
Mo1—C1	1.9640 (13)	C12—C13	1.388 (2)
Mo1—C2	1.9662 (15)	C12—H12	0.95
Mo1—C3	2.2794 (14)	C13—C14	1.384 (2)
Mo1—C9	2.3138 (14)	C13—H13	0.95
Mo1—C10	2.3216 (15)	C14—C15	1.382 (2)
Mo1—C8	2.3612 (14)	C14—H14	0.95
Mo1—C6	2.3795 (15)	C15—C16	1.3945 (19)
Mo1—C7	2.3805 (15)	C15—H15	0.95
P1—C17	1.8270 (12)	C16—H16	0.95
P1—C23	1.8289 (13)	C17—C18	1.3956 (17)
P1—C11	1.8341 (13)	C17—C22	1.3961 (18)
O1—C1	1.1563 (17)	C18—C19	1.3910 (18)
O2—C2	1.1555 (18)	C18—H18	0.95
O3—C3	1.2083 (19)	C19—C20	1.387 (2)
C3—C4	1.533 (2)	C19—H19	0.95
C4—C5	1.514 (2)	C20—C21	1.384 (2)
C4—H4A	0.99	C20—H20	0.95
C4—H4B	0.99	C21—C22	1.3909 (19)
C5—H5A	0.98	C21—H21	0.95
C5—H5B	0.98	C22—H22	0.95
C5—H5C	0.98	C23—C28	1.3907 (18)
C6—C7	1.408 (2)	C23—C24	1.3984 (18)
C6—C10	1.414 (3)	C24—C25	1.3814 (19)
C6—H6	1.0	C24—H24	0.95
C7—C8	1.409 (2)	C25—C26	1.392 (2)
C7—H7	1.0	C25—H25	0.95
C8—C9	1.409 (2)	C26—C27	1.376 (2)
C8—H8	1.0	C26—H26	0.95
C9—C10	1.422 (2)	C27—C28	1.398 (2)
C9—H9	1.0	C27—H27	0.95
C10—H10	1.0	C28—H28	0.95
C11—C16	1.3886 (18)		
			
C1—Mo1—C2	107.83 (6)	C7—C8—Mo1	73.47 (8)
C1—Mo1—C3	74.37 (5)	C9—C8—Mo1	70.63 (8)
C2—Mo1—C3	72.47 (5)	C7—C8—H8	125.9
C1—Mo1—C9	136.85 (6)	C9—C8—H8	125.9
C2—Mo1—C9	100.67 (6)	Mo1—C8—H8	125.9
C3—Mo1—C9	84.47 (6)	C8—C9—C10	107.75 (14)
C1—Mo1—C10	103.82 (6)	C8—C9—Mo1	74.31 (8)
C2—Mo1—C10	133.28 (6)	C10—C9—Mo1	72.43 (8)
C3—Mo1—C10	84.30 (6)	C8—C9—H9	125.8
C9—Mo1—C10	35.73 (6)	C10—C9—H9	125.8
C1—Mo1—C8	155.48 (6)	Mo1—C9—H9	125.8
C2—Mo1—C8	96.57 (6)	C6—C10—C9	108.10 (15)
C3—Mo1—C8	116.63 (6)	C6—C10—Mo1	74.76 (9)
C9—Mo1—C8	35.06 (6)	C9—C10—Mo1	71.83 (9)
C10—Mo1—C8	58.45 (6)	C6—C10—H10	125.7
C1—Mo1—C6	97.82 (6)	C9—C10—H10	125.7
C2—Mo1—C6	154.34 (6)	Mo1—C10—H10	125.7
C3—Mo1—C6	116.12 (6)	C16—C11—C12	118.96 (12)
C9—Mo1—C6	58.55 (6)	C16—C11—P1	123.40 (10)
C10—Mo1—C6	34.97 (6)	C12—C11—P1	117.64 (10)
C8—Mo1—C6	57.79 (6)	C13—C12—C11	120.73 (14)
C1—Mo1—C7	123.06 (6)	C13—C12—H12	119.6
C2—Mo1—C7	123.54 (6)	C11—C12—H12	119.6
C3—Mo1—C7	140.20 (6)	C14—C13—C12	119.95 (14)
C9—Mo1—C7	58.03 (6)	C14—C13—H13	120.0
C10—Mo1—C7	57.85 (6)	C12—C13—H13	120.0
C8—Mo1—C7	34.56 (6)	C15—C14—C13	119.77 (13)
C6—Mo1—C7	34.42 (6)	C15—C14—H14	120.1
C1—Mo1—P1	79.34 (4)	C13—C14—H14	120.1
C2—Mo1—P1	78.28 (4)	C14—C15—C16	120.48 (14)
C3—Mo1—P1	131.76 (4)	C14—C15—H15	119.8
C9—Mo1—P1	139.01 (4)	C16—C15—H15	119.8
C10—Mo1—P1	141.74 (5)	C11—C16—C15	120.10 (13)
C8—Mo1—P1	103.97 (4)	C11—C16—H16	119.9
C6—Mo1—P1	106.91 (4)	C15—C16—H16	119.9
C7—Mo1—P1	88.01 (4)	C18—C17—C22	118.98 (12)
C17—P1—C23	102.95 (6)	C18—C17—P1	119.14 (9)
C17—P1—C11	102.96 (6)	C22—C17—P1	121.83 (10)
C23—P1—C11	103.95 (6)	C19—C18—C17	120.29 (12)
C17—P1—Mo1	119.32 (4)	C19—C18—H18	119.9
C23—P1—Mo1	111.87 (4)	C17—C18—H18	119.9
C11—P1—Mo1	114.09 (4)	C20—C19—C18	120.26 (13)
O1—C1—Mo1	175.81 (12)	C20—C19—H19	119.9
O2—C2—Mo1	176.43 (13)	C18—C19—H19	119.9
O3—C3—C4	118.46 (14)	C21—C20—C19	119.83 (13)
O3—C3—Mo1	119.78 (12)	C21—C20—H20	120.1
C4—C3—Mo1	121.76 (10)	C19—C20—H20	120.1
C5—C4—C3	115.02 (15)	C20—C21—C22	120.23 (13)
C5—C4—H4A	108.5	C20—C21—H21	119.9
C3—C4—H4A	108.5	C22—C21—H21	119.9
C5—C4—H4B	108.5	C21—C22—C17	120.39 (12)
C3—C4—H4B	108.5	C21—C22—H22	119.8
H4A—C4—H4B	107.5	C17—C22—H22	119.8
C4—C5—H5A	109.5	C28—C23—C24	119.14 (12)
C4—C5—H5B	109.5	C28—C23—P1	122.88 (10)
H5A—C5—H5B	109.5	C24—C23—P1	117.61 (10)
C4—C5—H5C	109.5	C25—C24—C23	120.42 (13)
H5A—C5—H5C	109.5	C25—C24—H24	119.8
H5B—C5—H5C	109.5	C23—C24—H24	119.8
C7—C6—C10	107.44 (14)	C24—C25—C26	120.02 (14)
C7—C6—Mo1	72.83 (9)	C24—C25—H25	120.0
C10—C6—Mo1	70.27 (9)	C26—C25—H25	120.0
C7—C6—H6	126.2	C27—C26—C25	120.14 (13)
C10—C6—H6	126.2	C27—C26—H26	119.9
Mo1—C6—H6	126.2	C25—C26—H26	119.9
C6—C7—C8	108.81 (15)	C26—C27—C28	120.10 (13)
C6—C7—Mo1	72.75 (9)	C26—C27—H27	120.0
C8—C7—Mo1	71.97 (8)	C28—C27—H27	120.0
C6—C7—H7	125.5	C23—C28—C27	120.16 (13)
C8—C7—H7	125.5	C23—C28—H28	119.9
Mo1—C7—H7	125.5	C27—C28—H28	119.9
C7—C8—C9	107.89 (14)		
			
O3—C3—C4—C5	−5.3 (2)	C14—C15—C16—C11	0.7 (2)
Mo1—C3—C4—C5	175.31 (12)	C23—P1—C17—C18	157.21 (10)
C10—C6—C7—C8	−1.24 (17)	C11—P1—C17—C18	−94.93 (11)
Mo1—C6—C7—C8	−63.38 (11)	Mo1—P1—C17—C18	32.63 (12)
C10—C6—C7—Mo1	62.13 (10)	C23—P1—C17—C22	−25.50 (12)
C6—C7—C8—C9	1.22 (17)	C11—P1—C17—C22	82.36 (11)
Mo1—C7—C8—C9	−62.66 (10)	Mo1—P1—C17—C22	−150.08 (9)
C6—C7—C8—Mo1	63.88 (11)	C22—C17—C18—C19	−1.5 (2)
C7—C8—C9—C10	−0.72 (17)	P1—C17—C18—C19	175.84 (11)
Mo1—C8—C9—C10	−65.22 (10)	C17—C18—C19—C20	1.6 (2)
C7—C8—C9—Mo1	64.51 (11)	C18—C19—C20—C21	−0.3 (2)
C7—C6—C10—C9	0.79 (17)	C19—C20—C21—C22	−1.2 (2)
Mo1—C6—C10—C9	64.59 (11)	C20—C21—C22—C17	1.3 (2)
C7—C6—C10—Mo1	−63.80 (10)	C18—C17—C22—C21	0.1 (2)
C8—C9—C10—C6	−0.05 (17)	P1—C17—C22—C21	−177.21 (10)
Mo1—C9—C10—C6	−66.52 (10)	C17—P1—C23—C28	118.22 (11)
C8—C9—C10—Mo1	66.48 (11)	C11—P1—C23—C28	11.11 (13)
C17—P1—C11—C16	−3.65 (12)	Mo1—P1—C23—C28	−112.46 (11)
C23—P1—C11—C16	103.45 (12)	C17—P1—C23—C24	−68.86 (11)
Mo1—P1—C11—C16	−134.44 (10)	C11—P1—C23—C24	−175.97 (10)
C17—P1—C11—C12	175.23 (11)	Mo1—P1—C23—C24	60.46 (11)
C23—P1—C11—C12	−77.66 (12)	C28—C23—C24—C25	1.1 (2)
Mo1—P1—C11—C12	44.44 (12)	P1—C23—C24—C25	−172.08 (11)
C16—C11—C12—C13	0.2 (2)	C23—C24—C25—C26	−0.2 (2)
P1—C11—C12—C13	−178.74 (12)	C24—C25—C26—C27	−0.7 (2)
C11—C12—C13—C14	0.5 (2)	C25—C26—C27—C28	0.8 (2)
C12—C13—C14—C15	−0.6 (2)	C24—C23—C28—C27	−1.1 (2)
C13—C14—C15—C16	0.0 (2)	P1—C23—C28—C27	171.76 (11)
C12—C11—C16—C15	−0.8 (2)	C26—C27—C28—C23	0.1 (2)
P1—C11—C16—C15	178.09 (10)		

### Dicarbonyl(η5-cyclopentadienyl)propionyl(triphenylphosphane-κP)molybdenum(II) (1) Hydrogen-bond geometry (Å, º)

**Table:**

*D*—H···*A*	*D*—H	H···*A*	*D*···*A*	*D*—H···*A*
C12—H12···O2^i^	0.95	2.36	3.1188 (18)	137
C6—H6···O1^ii^	1.00	2.60	3.545 (2)	158

Symmetry codes: (i) −*x*+1, −*y*+1, −*z*+1; (ii) −*x*, −*y*+1, −*z*.

### Dicarbonyl(η5-cyclopentadienyl)propionyl[tris(4-fluorophenyl)phosphane-κP]molybdenum(II) (2) Crystal data

**Table:**

[Mo(C_5_H_5_)(C_3_H_5_O)(C_18_H_12_F_3_P)(CO)_2_]	*F*(000) = 2384
*M**_r_* = 590.36	*D*_x_ = 1.595 Mg m^−^^3^
Monoclinic, *P*2_1_/*c*	Mo *K*α radiation, λ = 0.71073 Å
*a* = 11.7991 (4) Å	Cell parameters from 9718 reflections
*b* = 18.6907 (8) Å	θ = 2.3–30.5°
*c* = 22.4744 (8) Å	µ = 0.65 mm^−^^1^
β = 97.256 (2)°	*T* = 170 K
*V* = 4916.7 (3) Å^3^	Prism, yellow
*Z* = 8	0.05 × 0.05 × 0.05 mm

### Dicarbonyl(η5-cyclopentadienyl)propionyl[tris(4-fluorophenyl)phosphane-κP]molybdenum(II) (2) Data collection

**Table:**

Bruker D8 QUEST ECO diffractometer	10047 independent reflections
Radiation source: sealed tube, Siemens KFFMO2K-90C	8547 reflections with *I* > 2σ(*I*)
Curved Graphite monochromator	*R*_int_ = 0.042
Detector resolution: 7.3910 pixels mm^-1^	θ_max_ = 26.4°, θ_min_ = 2.3°
φ and ω scans	*h* = −14→14
Absorption correction: multi-scan (Krause *et al.*, 2015)	*k* = −23→23
*T*_min_ = 0.86, *T*_max_ = 0.97	*l* = −28→27
73758 measured reflections	

### Dicarbonyl(η5-cyclopentadienyl)propionyl[tris(4-fluorophenyl)phosphane-κP]molybdenum(II) (2) Refinement

**Table:**

Refinement on *F*^2^	Primary atom site location: dual
Least-squares matrix: full	Secondary atom site location: difference Fourier map
*R*[*F*^2^ > 2σ(*F*^2^)] = 0.029	Hydrogen site location: inferred from neighbouring sites
*wR*(*F*^2^) = 0.063	H-atom parameters constrained
*S* = 1.07	*w* = 1/[σ^2^(*F*_o_^2^) + (0.0211*P*)^2^ + 4.4865*P*] where *P* = (*F*_o_^2^ + 2*F*_c_^2^)/3
10047 reflections	(Δ/σ)_max_ = 0.002
651 parameters	Δρ_max_ = 0.37 e Å^−^^3^
0 restraints	Δρ_min_ = −0.39 e Å^−^^3^

### Dicarbonyl(η5-cyclopentadienyl)propionyl[tris(4-fluorophenyl)phosphane-κP]molybdenum(II) (2) Special details

**Table:**

Geometry. All esds (except the esd in the dihedral angle between two l.s. planes) are estimated using the full covariance matrix. The cell esds are taken into account individually in the estimation of esds in distances, angles and torsion angles; correlations between esds in cell parameters are only used when they are defined by crystal symmetry. An approximate (isotropic) treatment of cell esds is used for estimating esds involving l.s. planes.

### Dicarbonyl(η5-cyclopentadienyl)propionyl[tris(4-fluorophenyl)phosphane-κP]molybdenum(II) (2) Fractional atomic coordinates and isotropic or equivalent isotropic displacement parameters (Å^2^)

**Table:**

	*x*	*y*	*z*	*U*_iso_*/*U*_eq_	
Mo1	0.68359 (2)	0.16744 (2)	0.31097 (2)	0.01937 (5)	
P1	0.50728 (5)	0.18602 (3)	0.35791 (3)	0.01993 (12)	
F1	0.54908 (16)	0.08511 (12)	0.61078 (7)	0.0637 (6)	
F2	0.08121 (14)	0.03649 (10)	0.25717 (8)	0.0533 (5)	
F3	0.36509 (14)	0.49064 (7)	0.35239 (8)	0.0441 (4)	
O1	0.51071 (16)	0.18266 (10)	0.19490 (8)	0.0399 (5)	
O2	0.74633 (16)	0.31199 (10)	0.37628 (8)	0.0361 (4)	
O3	0.88132 (15)	0.22239 (10)	0.24695 (8)	0.0333 (4)	
C1	0.5734 (2)	0.18193 (12)	0.23876 (11)	0.0256 (5)	
C2	0.71837 (19)	0.25955 (13)	0.35133 (10)	0.0243 (5)	
C3	0.7827 (2)	0.23663 (13)	0.25283 (10)	0.0248 (5)	
C4	0.7294 (2)	0.30324 (14)	0.22084 (12)	0.0346 (6)	
H4A	0.66605	0.28786	0.190403	0.041*	
H4B	0.696117	0.333204	0.250547	0.041*	
C5	0.8111 (3)	0.34882 (15)	0.19021 (13)	0.0409 (7)	
H5A	0.84251	0.32037	0.159538	0.061*	
H5B	0.873425	0.365392	0.219977	0.061*	
H5C	0.77008	0.3902	0.171316	0.061*	
C6	0.6987 (3)	0.04365 (14)	0.29401 (13)	0.0414 (7)	
H6	0.647429	0.014715	0.264404	0.05*	
C7	0.6872 (2)	0.05233 (13)	0.35502 (13)	0.0345 (6)	
H7	0.625273	0.031156	0.376022	0.041*	
C8	0.7818 (2)	0.09086 (14)	0.38260 (12)	0.0345 (6)	
H8	0.799791	0.100797	0.426502	0.041*	
C9	0.8523 (2)	0.10673 (15)	0.33829 (12)	0.0368 (6)	
H9	0.930101	0.128742	0.345501	0.044*	
C10	0.8008 (3)	0.07731 (15)	0.28325 (13)	0.0421 (7)	
H10	0.835835	0.075189	0.245032	0.05*	
C11	0.5180 (2)	0.15457 (12)	0.43586 (10)	0.0236 (5)	
C12	0.6089 (2)	0.17724 (15)	0.47718 (12)	0.0350 (6)	
H12	0.664562	0.208646	0.464597	0.042*	
C13	0.6194 (2)	0.15470 (18)	0.53629 (12)	0.0435 (7)	
H13	0.680534	0.171027	0.564616	0.052*	
C14	0.5390 (2)	0.10810 (17)	0.55288 (12)	0.0408 (7)	
C15	0.4497 (2)	0.08329 (14)	0.51394 (12)	0.0354 (6)	
H15	0.395996	0.050678	0.526812	0.043*	
C16	0.4392 (2)	0.10706 (13)	0.45480 (11)	0.0278 (5)	
H16	0.377406	0.090595	0.426987	0.033*	
C17	0.3749 (2)	0.14313 (12)	0.32379 (10)	0.0238 (5)	
C18	0.3798 (2)	0.07402 (13)	0.30120 (11)	0.0312 (6)	
H18	0.451868	0.051639	0.300493	0.037*	
C19	0.2808 (2)	0.03741 (15)	0.27971 (12)	0.0363 (6)	
H19	0.284022	−0.010424	0.265731	0.044*	
C20	0.1781 (2)	0.07191 (16)	0.27914 (12)	0.0360 (6)	
C21	0.1688 (2)	0.14034 (15)	0.29969 (11)	0.0321 (6)	
H21	0.096469	0.163162	0.297831	0.039*	
C22	0.2682 (2)	0.17545 (13)	0.32338 (11)	0.0275 (5)	
H22	0.263447	0.222164	0.33953	0.033*	
C23	0.46301 (18)	0.27942 (12)	0.36021 (10)	0.0212 (5)	
C24	0.4234 (2)	0.31163 (12)	0.30539 (11)	0.0270 (5)	
H24	0.421085	0.284722	0.269401	0.032*	
C25	0.3877 (2)	0.38197 (13)	0.30282 (12)	0.0318 (6)	
H25	0.358032	0.403262	0.265723	0.038*	
C26	0.3961 (2)	0.42019 (12)	0.35498 (13)	0.0308 (6)	
C27	0.4348 (2)	0.39122 (13)	0.40983 (12)	0.0306 (6)	
H27	0.439161	0.419233	0.445318	0.037*	
C28	0.4673 (2)	0.31977 (13)	0.41208 (11)	0.0255 (5)	
H28	0.492908	0.298322	0.449712	0.031*	
Mo2	0.13778 (2)	0.20503 (2)	0.57103 (2)	0.01918 (5)	
P2	0.07626 (5)	0.32692 (3)	0.53987 (3)	0.01888 (12)	
F4	−0.41095 (12)	0.38483 (9)	0.56536 (7)	0.0443 (4)	
F5	0.36489 (13)	0.55112 (8)	0.67019 (7)	0.0405 (4)	
F6	0.08738 (15)	0.40659 (9)	0.28469 (7)	0.0483 (4)	
O4	−0.11576 (15)	0.17823 (10)	0.51826 (9)	0.0389 (4)	
O5	0.28370 (16)	0.23246 (10)	0.46697 (9)	0.0408 (5)	
O6	0.1843 (2)	0.04766 (10)	0.54648 (9)	0.0584 (6)	
C29	−0.0209 (2)	0.18824 (12)	0.53604 (11)	0.0250 (5)	
C30	0.2268 (2)	0.22200 (12)	0.50419 (11)	0.0261 (5)	
C31	0.1403 (2)	0.09868 (12)	0.52100 (11)	0.0265 (5)	
C32	0.0920 (3)	0.08959 (15)	0.45594 (12)	0.0392 (7)	
H32A	0.008542	0.097943	0.452191	0.047*	
H32B	0.125178	0.127064	0.43224	0.047*	
C33	0.1125 (3)	0.01792 (15)	0.42855 (13)	0.0441 (7)	
H33A	0.073311	0.016208	0.387439	0.066*	
H33B	0.082777	−0.02003	0.452393	0.066*	
H33C	0.194657	0.010948	0.428005	0.066*	
C34	0.2910 (2)	0.21959 (17)	0.64802 (12)	0.0412 (7)	
H34	0.365723	0.243055	0.64373	0.049*	
C35	0.1968 (3)	0.25274 (16)	0.66799 (11)	0.0433 (7)	
H35	0.192359	0.30424	0.679536	0.052*	
C36	0.1135 (3)	0.20086 (17)	0.67425 (11)	0.0408 (7)	
H36	0.040367	0.208562	0.691483	0.049*	
C37	0.1563 (2)	0.13425 (15)	0.65645 (11)	0.0373 (6)	
H37	0.120101	0.086396	0.660455	0.045*	
C38	0.2670 (2)	0.14677 (15)	0.64067 (11)	0.0358 (6)	
H38	0.322423	0.109088	0.631453	0.043*	
C39	−0.07161 (19)	0.35126 (12)	0.54894 (10)	0.0202 (5)	
C40	−0.1144 (2)	0.33622 (13)	0.60274 (11)	0.0293 (5)	
H40	−0.064987	0.31691	0.63546	0.035*	
C41	−0.2281 (2)	0.34908 (14)	0.60907 (11)	0.0314 (6)	
H41	−0.256614	0.339932	0.646011	0.038*	
C42	−0.2982 (2)	0.37531 (13)	0.56070 (12)	0.0290 (5)	
C43	−0.2598 (2)	0.39207 (14)	0.50748 (11)	0.0314 (6)	
H43	−0.309978	0.411534	0.475153	0.038*	
C44	−0.1453 (2)	0.37987 (13)	0.50190 (11)	0.0264 (5)	
H44	−0.11709	0.391315	0.465296	0.032*	
C45	0.16211 (19)	0.39779 (12)	0.57976 (10)	0.0221 (5)	
C46	0.1136 (2)	0.45612 (12)	0.60505 (10)	0.0243 (5)	
H46	0.032784	0.460151	0.601914	0.029*	
C47	0.1819 (2)	0.50884 (13)	0.63496 (11)	0.0279 (5)	
H47	0.148881	0.548962	0.652204	0.034*	
C48	0.2979 (2)	0.50132 (13)	0.63887 (11)	0.0285 (5)	
C49	0.3500 (2)	0.44497 (13)	0.61377 (11)	0.0298 (6)	
H49	0.430847	0.44186	0.616714	0.036*	
C50	0.2813 (2)	0.39324 (13)	0.58426 (11)	0.0274 (5)	
H50	0.315441	0.35379	0.566651	0.033*	
C51	0.08310 (18)	0.35142 (12)	0.46138 (10)	0.0204 (5)	
C52	0.1267 (2)	0.41698 (12)	0.44605 (11)	0.0250 (5)	
H52	0.155534	0.449472	0.476796	0.03*	
C53	0.1287 (2)	0.43575 (14)	0.38613 (11)	0.0297 (5)	
H53	0.158918	0.480455	0.375637	0.036*	
C54	0.0860 (2)	0.38815 (15)	0.34296 (11)	0.0316 (6)	
C55	0.0410 (2)	0.32290 (14)	0.35584 (11)	0.0322 (6)	
H55	0.011264	0.291174	0.324697	0.039*	
C56	0.0404 (2)	0.30477 (13)	0.41563 (11)	0.0269 (5)	
H56	0.010257	0.259799	0.425552	0.032*	

### Dicarbonyl(η5-cyclopentadienyl)propionyl[tris(4-fluorophenyl)phosphane-κP]molybdenum(II) (2) Atomic displacement parameters (Å^2^)

**Table:**

	*U*^11^	*U*^22^	*U*^33^	*U*^12^	*U*^13^	*U*^23^
Mo1	0.02038 (10)	0.01882 (10)	0.01827 (10)	0.00364 (8)	−0.00004 (7)	−0.00050 (7)
P1	0.0203 (3)	0.0190 (3)	0.0200 (3)	0.0007 (2)	0.0005 (2)	0.0010 (2)
F1	0.0570 (12)	0.1040 (16)	0.0297 (9)	−0.0035 (11)	0.0034 (8)	0.0313 (10)
F2	0.0360 (9)	0.0682 (12)	0.0554 (11)	−0.0274 (9)	0.0049 (8)	−0.0178 (9)
F3	0.0454 (10)	0.0205 (7)	0.0669 (11)	0.0049 (7)	0.0087 (8)	−0.0037 (7)
O1	0.0374 (11)	0.0506 (12)	0.0284 (10)	0.0065 (9)	−0.0093 (8)	−0.0035 (9)
O2	0.0375 (10)	0.0331 (10)	0.0377 (10)	−0.0074 (8)	0.0049 (8)	−0.0128 (8)
O3	0.0276 (9)	0.0392 (10)	0.0341 (10)	0.0025 (8)	0.0079 (8)	0.0000 (8)
C1	0.0268 (12)	0.0247 (12)	0.0254 (12)	0.0038 (10)	0.0037 (10)	−0.0031 (10)
C2	0.0199 (11)	0.0290 (13)	0.0243 (12)	−0.0001 (10)	0.0042 (9)	0.0002 (10)
C3	0.0287 (13)	0.0271 (12)	0.0183 (11)	−0.0015 (10)	0.0015 (10)	−0.0039 (9)
C4	0.0399 (15)	0.0330 (14)	0.0316 (14)	0.0057 (12)	0.0075 (12)	0.0039 (11)
C5	0.0494 (17)	0.0356 (15)	0.0381 (16)	−0.0021 (13)	0.0073 (13)	0.0064 (12)
C6	0.0561 (19)	0.0191 (12)	0.0470 (17)	0.0114 (12)	−0.0009 (14)	−0.0039 (12)
C7	0.0371 (15)	0.0207 (12)	0.0456 (16)	0.0074 (11)	0.0049 (12)	0.0096 (11)
C8	0.0336 (14)	0.0354 (14)	0.0326 (14)	0.0134 (12)	−0.0024 (11)	0.0123 (12)
C9	0.0254 (13)	0.0428 (15)	0.0415 (16)	0.0161 (12)	0.0012 (12)	0.0133 (13)
C10	0.0544 (18)	0.0326 (14)	0.0414 (16)	0.0265 (14)	0.0146 (14)	0.0023 (12)
C11	0.0242 (12)	0.0254 (12)	0.0216 (11)	0.0050 (10)	0.0042 (9)	0.0033 (9)
C12	0.0266 (13)	0.0496 (16)	0.0284 (13)	−0.0042 (12)	0.0019 (11)	0.0058 (12)
C13	0.0318 (15)	0.071 (2)	0.0255 (14)	−0.0025 (14)	−0.0042 (11)	0.0077 (14)
C14	0.0394 (16)	0.0586 (19)	0.0249 (14)	0.0102 (14)	0.0065 (12)	0.0149 (13)
C15	0.0370 (15)	0.0355 (14)	0.0356 (15)	0.0022 (12)	0.0118 (12)	0.0130 (12)
C16	0.0280 (13)	0.0244 (12)	0.0311 (13)	0.0041 (10)	0.0048 (10)	0.0020 (10)
C17	0.0244 (12)	0.0249 (12)	0.0215 (12)	−0.0048 (10)	0.0007 (9)	0.0028 (9)
C18	0.0315 (14)	0.0293 (13)	0.0322 (14)	−0.0013 (11)	0.0019 (11)	−0.0038 (11)
C19	0.0417 (16)	0.0345 (14)	0.0324 (14)	−0.0108 (12)	0.0038 (12)	−0.0077 (12)
C20	0.0312 (14)	0.0508 (17)	0.0261 (13)	−0.0182 (13)	0.0040 (11)	−0.0026 (12)
C21	0.0244 (13)	0.0432 (15)	0.0292 (14)	−0.0054 (11)	0.0052 (10)	0.0013 (12)
C22	0.0278 (13)	0.0289 (13)	0.0260 (12)	−0.0027 (10)	0.0040 (10)	0.0026 (10)
C23	0.0159 (10)	0.0212 (11)	0.0263 (12)	−0.0006 (9)	0.0014 (9)	0.0002 (9)
C24	0.0317 (13)	0.0225 (12)	0.0260 (12)	0.0020 (10)	0.0004 (10)	−0.0006 (10)
C25	0.0342 (14)	0.0243 (12)	0.0355 (14)	0.0018 (11)	−0.0012 (11)	0.0053 (11)
C26	0.0216 (12)	0.0187 (11)	0.0526 (17)	0.0007 (10)	0.0071 (11)	−0.0025 (11)
C27	0.0273 (13)	0.0311 (13)	0.0345 (14)	−0.0038 (11)	0.0084 (11)	−0.0106 (11)
C28	0.0223 (12)	0.0289 (12)	0.0255 (12)	−0.0004 (10)	0.0039 (10)	−0.0013 (10)
Mo2	0.01832 (10)	0.02103 (10)	0.01787 (10)	0.00077 (8)	0.00110 (7)	0.00333 (8)
P2	0.0180 (3)	0.0205 (3)	0.0182 (3)	0.0014 (2)	0.0026 (2)	0.0011 (2)
F4	0.0207 (7)	0.0672 (11)	0.0468 (10)	0.0075 (7)	0.0113 (7)	−0.0050 (8)
F5	0.0333 (8)	0.0364 (8)	0.0515 (10)	−0.0146 (7)	0.0046 (7)	−0.0119 (7)
F6	0.0641 (11)	0.0604 (11)	0.0221 (8)	0.0108 (9)	0.0129 (8)	0.0110 (7)
O4	0.0235 (10)	0.0388 (11)	0.0529 (12)	−0.0036 (8)	−0.0007 (9)	−0.0067 (9)
O5	0.0381 (11)	0.0421 (11)	0.0470 (12)	0.0118 (9)	0.0244 (9)	0.0136 (9)
O6	0.0987 (19)	0.0270 (10)	0.0434 (12)	0.0110 (11)	−0.0143 (12)	0.0041 (9)
C29	0.0267 (13)	0.0209 (11)	0.0277 (12)	0.0018 (10)	0.0050 (10)	0.0014 (10)
C30	0.0238 (12)	0.0227 (12)	0.0314 (13)	0.0052 (10)	0.0019 (11)	0.0025 (10)
C31	0.0251 (12)	0.0227 (12)	0.0316 (13)	−0.0020 (10)	0.0035 (10)	0.0035 (10)
C32	0.0474 (17)	0.0379 (15)	0.0309 (14)	0.0088 (13)	−0.0011 (12)	−0.0035 (12)
C33	0.0590 (19)	0.0355 (15)	0.0383 (16)	−0.0063 (14)	0.0078 (14)	−0.0056 (13)
C34	0.0361 (15)	0.0618 (19)	0.0225 (13)	−0.0127 (14)	−0.0090 (11)	0.0028 (13)
C35	0.073 (2)	0.0383 (15)	0.0160 (12)	0.0004 (15)	−0.0068 (13)	−0.0004 (11)
C36	0.0420 (16)	0.0634 (19)	0.0181 (12)	0.0140 (15)	0.0072 (11)	0.0112 (13)
C37	0.0483 (17)	0.0379 (15)	0.0239 (13)	−0.0049 (13)	−0.0024 (12)	0.0147 (11)
C38	0.0373 (15)	0.0450 (16)	0.0229 (13)	0.0143 (13)	−0.0048 (11)	0.0066 (11)
C39	0.0201 (11)	0.0202 (11)	0.0200 (11)	0.0019 (9)	0.0009 (9)	−0.0004 (9)
C40	0.0292 (13)	0.0348 (14)	0.0243 (12)	0.0049 (11)	0.0045 (10)	0.0052 (11)
C41	0.0308 (13)	0.0384 (14)	0.0274 (13)	0.0050 (11)	0.0123 (11)	0.0046 (11)
C42	0.0196 (12)	0.0323 (13)	0.0363 (14)	0.0009 (10)	0.0082 (10)	−0.0070 (11)
C43	0.0233 (12)	0.0424 (15)	0.0276 (13)	0.0074 (11)	−0.0004 (10)	0.0001 (11)
C44	0.0233 (12)	0.0347 (13)	0.0216 (12)	0.0027 (10)	0.0044 (10)	0.0017 (10)
C45	0.0236 (12)	0.0235 (11)	0.0189 (11)	−0.0017 (9)	0.0010 (9)	0.0028 (9)
C46	0.0210 (11)	0.0263 (12)	0.0248 (12)	0.0019 (10)	0.0005 (9)	0.0010 (10)
C47	0.0299 (13)	0.0233 (12)	0.0311 (13)	0.0022 (10)	0.0057 (11)	−0.0022 (10)
C48	0.0283 (13)	0.0256 (12)	0.0305 (13)	−0.0086 (10)	−0.0003 (10)	0.0010 (10)
C49	0.0208 (12)	0.0305 (13)	0.0383 (15)	−0.0027 (10)	0.0046 (11)	0.0028 (11)
C50	0.0223 (12)	0.0277 (12)	0.0329 (13)	0.0021 (10)	0.0063 (10)	−0.0027 (10)
C51	0.0187 (11)	0.0242 (11)	0.0183 (11)	0.0051 (9)	0.0029 (9)	0.0011 (9)
C52	0.0250 (12)	0.0252 (12)	0.0254 (12)	0.0026 (10)	0.0053 (10)	0.0015 (10)
C53	0.0299 (13)	0.0314 (13)	0.0295 (13)	0.0052 (11)	0.0109 (11)	0.0076 (11)
C54	0.0319 (14)	0.0434 (15)	0.0215 (12)	0.0143 (12)	0.0115 (10)	0.0088 (11)
C55	0.0360 (14)	0.0364 (14)	0.0232 (12)	0.0096 (12)	0.0001 (11)	−0.0033 (11)
C56	0.0307 (13)	0.0244 (12)	0.0253 (12)	0.0033 (10)	0.0023 (10)	0.0014 (10)

### Dicarbonyl(η5-cyclopentadienyl)propionyl[tris(4-fluorophenyl)phosphane-κP]molybdenum(II) (2) Geometric parameters (Å, º)

**Table:**

Mo1—P1	2.4730 (6)	Mo2—C29	1.962 (2)
Mo1—C1	1.966 (2)	Mo2—C30	1.964 (3)
Mo1—C2	1.965 (2)	Mo2—C31	2.286 (2)
Mo1—C3	2.265 (2)	Mo2—C38	2.315 (2)
Mo1—C9	2.306 (2)	Mo2—C37	2.319 (2)
Mo1—C10	2.314 (2)	Mo2—C34	2.355 (3)
Mo1—C8	2.348 (2)	Mo2—C36	2.374 (3)
Mo1—C6	2.355 (3)	Mo2—C35	2.376 (3)
Mo1—C7	2.366 (2)	Mo2—P2	2.4654 (6)
P1—C23	1.825 (2)	P2—C45	1.832 (2)
P1—C17	1.835 (2)	P2—C51	1.834 (2)
P1—C11	1.837 (2)	P2—C39	1.839 (2)
F1—C14	1.361 (3)	F4—C42	1.359 (3)
F2—C20	1.359 (3)	F5—C48	1.359 (3)
F3—C26	1.366 (3)	F6—C54	1.356 (3)
O1—C1	1.155 (3)	O4—C29	1.155 (3)
O2—C2	1.156 (3)	O5—C30	1.154 (3)
O3—C3	1.218 (3)	O6—C31	1.196 (3)
C3—C4	1.532 (3)	C31—C32	1.511 (3)
C4—C5	1.515 (4)	C32—C33	1.506 (4)
C4—H4A	0.99	C32—H32A	0.99
C4—H4B	0.99	C32—H32B	0.99
C5—H5A	0.98	C33—H33A	0.98
C5—H5B	0.98	C33—H33B	0.98
C5—H5C	0.98	C33—H33C	0.98
C6—C7	1.404 (4)	C34—C35	1.396 (4)
C6—C10	1.406 (4)	C34—C38	1.396 (4)
C6—H6	1.0	C34—H34	1.0
C7—C8	1.405 (4)	C35—C36	1.400 (4)
C7—H7	1.0	C35—H35	1.0
C8—C9	1.408 (4)	C36—C37	1.420 (4)
C8—H8	1.0	C36—H36	1.0
C9—C10	1.419 (4)	C37—C38	1.416 (4)
C9—H9	1.0	C37—H37	1.0
C10—H10	1.0	C38—H38	1.0
C11—C16	1.391 (3)	C39—C44	1.388 (3)
C11—C12	1.393 (3)	C39—C40	1.397 (3)
C12—C13	1.384 (4)	C40—C41	1.388 (3)
C12—H12	0.95	C40—H40	0.95
C13—C14	1.373 (4)	C41—C42	1.371 (4)
C13—H13	0.95	C41—H41	0.95
C14—C15	1.364 (4)	C42—C43	1.368 (3)
C15—C16	1.392 (3)	C43—C44	1.391 (3)
C15—H15	0.95	C43—H43	0.95
C16—H16	0.95	C44—H44	0.95
C17—C18	1.392 (3)	C45—C46	1.386 (3)
C17—C22	1.395 (3)	C45—C50	1.400 (3)
C18—C19	1.387 (4)	C46—C47	1.391 (3)
C18—H18	0.95	C46—H46	0.95
C19—C20	1.371 (4)	C47—C48	1.367 (3)
C19—H19	0.95	C47—H47	0.95
C20—C21	1.369 (4)	C48—C49	1.376 (3)
C21—C22	1.390 (3)	C49—C50	1.376 (3)
C21—H21	0.95	C49—H49	0.95
C22—H22	0.95	C50—H50	0.95
C23—C28	1.384 (3)	C51—C52	1.389 (3)
C23—C24	1.398 (3)	C51—C56	1.393 (3)
C24—C25	1.379 (3)	C52—C53	1.395 (3)
C24—H24	0.95	C52—H52	0.95
C25—C26	1.366 (4)	C53—C54	1.365 (4)
C25—H25	0.95	C53—H53	0.95
C26—C27	1.371 (4)	C54—C55	1.375 (4)
C27—C28	1.389 (3)	C55—C56	1.387 (3)
C27—H27	0.95	C55—H55	0.95
C28—H28	0.95	C56—H56	0.95
			
C1—Mo1—C2	109.77 (10)	C29—Mo2—C30	107.12 (10)
C1—Mo1—C3	77.34 (9)	C29—Mo2—C31	74.64 (9)
C2—Mo1—C3	70.93 (9)	C30—Mo2—C31	73.89 (9)
C2—Mo1—C9	100.67 (10)	C29—Mo2—C38	135.20 (10)
C1—Mo1—C9	138.27 (10)	C30—Mo2—C38	102.87 (10)
C3—Mo1—C9	86.76 (9)	C31—Mo2—C38	82.81 (9)
C2—Mo1—C10	131.68 (11)	C29—Mo2—C37	103.05 (10)
C1—Mo1—C10	103.50 (10)	C30—Mo2—C37	136.20 (10)
C3—Mo1—C10	83.74 (10)	C31—Mo2—C37	84.57 (9)
C9—Mo1—C10	35.76 (10)	C38—Mo2—C37	35.59 (10)
C2—Mo1—C8	99.28 (10)	C29—Mo2—C34	156.64 (10)
C1—Mo1—C8	150.20 (10)	C30—Mo2—C34	96.22 (10)
C3—Mo1—C8	119.84 (9)	C31—Mo2—C34	113.90 (10)
C9—Mo1—C8	35.20 (9)	C38—Mo2—C34	34.77 (10)
C10—Mo1—C8	58.73 (10)	C37—Mo2—C34	58.30 (10)
C2—Mo1—C6	156.88 (10)	C29—Mo2—C36	99.19 (10)
C1—Mo1—C6	93.30 (10)	C30—Mo2—C36	153.46 (10)
C3—Mo1—C6	114.34 (10)	C31—Mo2—C36	117.57 (10)
C9—Mo1—C6	58.62 (11)	C38—Mo2—C36	58.30 (10)
C10—Mo1—C6	35.04 (10)	C37—Mo2—C36	35.19 (10)
C8—Mo1—C6	58.07 (10)	C34—Mo2—C36	57.46 (10)
C2—Mo1—C7	127.71 (10)	C29—Mo2—C35	125.58 (11)
C1—Mo1—C7	116.47 (10)	C30—Mo2—C35	121.19 (11)
C3—Mo1—C7	140.99 (9)	C31—Mo2—C35	139.20 (9)
C9—Mo1—C7	58.14 (10)	C38—Mo2—C35	57.64 (10)
C10—Mo1—C7	58.07 (10)	C37—Mo2—C35	57.91 (10)
C8—Mo1—C7	34.68 (9)	C34—Mo2—C35	34.31 (10)
C6—Mo1—C7	34.61 (10)	C36—Mo2—C35	34.28 (10)
C1—Mo1—P1	80.12 (7)	C29—Mo2—P2	78.59 (7)
C2—Mo1—P1	79.50 (7)	C30—Mo2—P2	78.48 (7)
C3—Mo1—P1	133.55 (6)	C31—Mo2—P2	133.28 (6)
C9—Mo1—P1	134.42 (7)	C38—Mo2—P2	140.53 (7)
C10—Mo1—P1	141.31 (8)	C37—Mo2—P2	139.10 (7)
C8—Mo1—P1	99.33 (7)	C34—Mo2—P2	105.85 (8)
C6—Mo1—P1	106.98 (8)	C36—Mo2—P2	103.91 (7)
C7—Mo1—P1	85.46 (7)	C35—Mo2—P2	87.44 (7)
C23—P1—C17	101.43 (10)	C45—P2—C51	101.62 (10)
C23—P1—C11	105.34 (11)	C45—P2—C39	103.92 (10)
C17—P1—C11	102.19 (11)	C51—P2—C39	101.68 (10)
C23—P1—Mo1	113.94 (7)	C45—P2—Mo2	113.86 (7)
C17—P1—Mo1	118.55 (8)	C51—P2—Mo2	117.18 (7)
C11—P1—Mo1	113.67 (8)	C39—P2—Mo2	116.52 (7)
O1—C1—Mo1	172.5 (2)	O4—C29—Mo2	176.6 (2)
O2—C2—Mo1	175.3 (2)	O5—C30—Mo2	176.6 (2)
O3—C3—C4	117.9 (2)	O6—C31—C32	117.5 (2)
O3—C3—Mo1	120.52 (18)	O6—C31—Mo2	119.32 (19)
C4—C3—Mo1	121.58 (17)	C32—C31—Mo2	123.17 (17)
C5—C4—C3	115.1 (2)	C33—C32—C31	115.8 (2)
C5—C4—H4A	108.5	C33—C32—H32A	108.3
C3—C4—H4A	108.5	C31—C32—H32A	108.3
C5—C4—H4B	108.5	C33—C32—H32B	108.3
C3—C4—H4B	108.5	C31—C32—H32B	108.3
H4A—C4—H4B	107.5	H32A—C32—H32B	107.4
C4—C5—H5A	109.5	C32—C33—H33A	109.5
C4—C5—H5B	109.5	C32—C33—H33B	109.5
H5A—C5—H5B	109.5	H33A—C33—H33B	109.5
C4—C5—H5C	109.5	C32—C33—H33C	109.5
H5A—C5—H5C	109.5	H33A—C33—H33C	109.5
H5B—C5—H5C	109.5	H33B—C33—H33C	109.5
C7—C6—C10	107.9 (3)	C35—C34—C38	108.2 (3)
C7—C6—Mo1	73.13 (14)	C35—C34—Mo2	73.66 (16)
C10—C6—Mo1	70.88 (15)	C38—C34—Mo2	71.04 (14)
C7—C6—H6	125.9	C35—C34—H34	125.7
C10—C6—H6	125.9	C38—C34—H34	125.7
Mo1—C6—H6	125.9	Mo2—C34—H34	125.7
C6—C7—C8	108.7 (3)	C34—C35—C36	108.8 (3)
C6—C7—Mo1	72.26 (14)	C34—C35—Mo2	72.03 (15)
C8—C7—Mo1	71.94 (14)	C36—C35—Mo2	72.80 (15)
C6—C7—H7	125.6	C34—C35—H35	125.5
C8—C7—H7	125.6	C36—C35—H35	125.5
Mo1—C7—H7	125.6	Mo2—C35—H35	125.5
C7—C8—C9	107.7 (3)	C35—C36—C37	107.5 (3)
C7—C8—Mo1	73.38 (14)	C35—C36—Mo2	72.92 (15)
C9—C8—Mo1	70.80 (14)	C37—C36—Mo2	70.28 (14)
C7—C8—H8	126.0	C35—C36—H36	126.2
C9—C8—H8	126.0	C37—C36—H36	126.2
Mo1—C8—H8	126.0	Mo2—C36—H36	126.2
C8—C9—C10	108.0 (3)	C38—C37—C36	107.3 (3)
C8—C9—Mo1	74.01 (14)	C38—C37—Mo2	72.04 (14)
C10—C9—Mo1	72.42 (14)	C36—C37—Mo2	74.53 (14)
C8—C9—H9	125.8	C38—C37—H37	126.0
C10—C9—H9	125.8	C36—C37—H37	126.0
Mo1—C9—H9	125.8	Mo2—C37—H37	126.0
C6—C10—C9	107.8 (3)	C34—C38—C37	108.1 (3)
C6—C10—Mo1	74.08 (15)	C34—C38—Mo2	74.19 (15)
C9—C10—Mo1	71.82 (14)	C37—C38—Mo2	72.36 (14)
C6—C10—H10	125.9	C34—C38—H38	125.7
C9—C10—H10	125.9	C37—C38—H38	125.7
Mo1—C10—H10	125.9	Mo2—C38—H38	125.7
C16—C11—C12	118.6 (2)	C44—C39—C40	118.5 (2)
C16—C11—P1	121.88 (18)	C44—C39—P2	121.51 (17)
C12—C11—P1	119.53 (18)	C40—C39—P2	119.86 (17)
C13—C12—C11	121.0 (2)	C41—C40—C39	120.9 (2)
C13—C12—H12	119.5	C41—C40—H40	119.5
C11—C12—H12	119.5	C39—C40—H40	119.5
C14—C13—C12	118.2 (3)	C42—C41—C40	118.4 (2)
C14—C13—H13	120.9	C42—C41—H41	120.8
C12—C13—H13	120.9	C40—C41—H41	120.8
F1—C14—C15	118.2 (3)	F4—C42—C43	118.2 (2)
F1—C14—C13	118.7 (3)	F4—C42—C41	119.0 (2)
C15—C14—C13	123.1 (2)	C43—C42—C41	122.8 (2)
C14—C15—C16	118.2 (2)	C42—C43—C44	118.3 (2)
C14—C15—H15	120.9	C42—C43—H43	120.8
C16—C15—H15	120.9	C44—C43—H43	120.8
C11—C16—C15	121.0 (2)	C39—C44—C43	121.1 (2)
C11—C16—H16	119.5	C39—C44—H44	119.5
C15—C16—H16	119.5	C43—C44—H44	119.5
C18—C17—C22	118.6 (2)	C46—C45—C50	118.8 (2)
C18—C17—P1	119.04 (18)	C46—C45—P2	122.54 (18)
C22—C17—P1	122.22 (18)	C50—C45—P2	118.68 (18)
C19—C18—C17	120.9 (2)	C45—C46—C47	120.7 (2)
C19—C18—H18	119.6	C45—C46—H46	119.6
C17—C18—H18	119.6	C47—C46—H46	119.6
C20—C19—C18	118.4 (2)	C48—C47—C46	118.2 (2)
C20—C19—H19	120.8	C48—C47—H47	120.9
C18—C19—H19	120.8	C46—C47—H47	120.9
F2—C20—C21	118.6 (3)	F5—C48—C47	118.4 (2)
F2—C20—C19	118.4 (3)	F5—C48—C49	118.4 (2)
C21—C20—C19	123.0 (2)	C47—C48—C49	123.2 (2)
C20—C21—C22	118.1 (2)	C48—C49—C50	118.0 (2)
C20—C21—H21	121.0	C48—C49—H49	121.0
C22—C21—H21	121.0	C50—C49—H49	121.0
C21—C22—C17	121.0 (2)	C49—C50—C45	121.1 (2)
C21—C22—H22	119.5	C49—C50—H50	119.4
C17—C22—H22	119.5	C45—C50—H50	119.4
C28—C23—C24	118.7 (2)	C52—C51—C56	118.7 (2)
C28—C23—P1	124.40 (18)	C52—C51—P2	121.43 (17)
C24—C23—P1	116.89 (17)	C56—C51—P2	119.85 (17)
C25—C24—C23	120.9 (2)	C51—C52—C53	120.9 (2)
C25—C24—H24	119.5	C51—C52—H52	119.6
C23—C24—H24	119.5	C53—C52—H52	119.6
C26—C25—C24	118.3 (2)	C54—C53—C52	118.2 (2)
C26—C25—H25	120.9	C54—C53—H53	120.9
C24—C25—H25	120.9	C52—C53—H53	120.9
C25—C26—F3	118.5 (2)	F6—C54—C53	118.2 (2)
C25—C26—C27	123.0 (2)	F6—C54—C55	118.7 (2)
F3—C26—C27	118.4 (2)	C53—C54—C55	123.1 (2)
C26—C27—C28	118.1 (2)	C54—C55—C56	118.0 (2)
C26—C27—H27	120.9	C54—C55—H55	121.0
C28—C27—H27	120.9	C56—C55—H55	121.0
C23—C28—C27	120.8 (2)	C55—C56—C51	121.1 (2)
C23—C28—H28	119.6	C55—C56—H56	119.4
C27—C28—H28	119.6	C51—C56—H56	119.4
			
O3—C3—C4—C5	−6.1 (3)	O6—C31—C32—C33	−4.5 (4)
Mo1—C3—C4—C5	172.61 (18)	Mo2—C31—C32—C33	173.63 (19)
C10—C6—C7—C8	−0.4 (3)	C38—C34—C35—C36	0.9 (3)
Mo1—C6—C7—C8	−63.15 (17)	Mo2—C34—C35—C36	63.96 (19)
C10—C6—C7—Mo1	62.78 (18)	C38—C34—C35—Mo2	−63.05 (18)
C6—C7—C8—C9	0.4 (3)	C34—C35—C36—C37	−1.3 (3)
Mo1—C7—C8—C9	−62.91 (17)	Mo2—C35—C36—C37	62.17 (18)
C6—C7—C8—Mo1	63.36 (18)	C34—C35—C36—Mo2	−63.47 (18)
C7—C8—C9—C10	−0.4 (3)	C35—C36—C37—C38	1.2 (3)
Mo1—C8—C9—C10	−64.96 (17)	Mo2—C36—C37—C38	65.08 (17)
C7—C8—C9—Mo1	64.60 (17)	C35—C36—C37—Mo2	−63.90 (18)
C7—C6—C10—C9	0.1 (3)	C35—C34—C38—C37	−0.2 (3)
Mo1—C6—C10—C9	64.39 (17)	Mo2—C34—C38—C37	−64.91 (17)
C7—C6—C10—Mo1	−64.24 (18)	C35—C34—C38—Mo2	64.75 (19)
C8—C9—C10—C6	0.1 (3)	C36—C37—C38—C34	−0.6 (3)
Mo1—C9—C10—C6	−65.88 (18)	Mo2—C37—C38—C34	66.12 (18)
C8—C9—C10—Mo1	66.01 (18)	C36—C37—C38—Mo2	−66.75 (17)
C23—P1—C11—C16	108.3 (2)	C45—P2—C39—C44	105.6 (2)
C17—P1—C11—C16	2.7 (2)	C51—P2—C39—C44	0.4 (2)
Mo1—P1—C11—C16	−126.24 (18)	Mo2—P2—C39—C44	−128.27 (18)
C23—P1—C11—C12	−73.3 (2)	C45—P2—C39—C40	−79.0 (2)
C17—P1—C11—C12	−178.9 (2)	C51—P2—C39—C40	175.70 (19)
Mo1—P1—C11—C12	52.1 (2)	Mo2—P2—C39—C40	47.1 (2)
C16—C11—C12—C13	−1.7 (4)	C44—C39—C40—C41	0.4 (4)
P1—C11—C12—C13	179.8 (2)	P2—C39—C40—C41	−175.1 (2)
C11—C12—C13—C14	1.4 (4)	C39—C40—C41—C42	1.6 (4)
C12—C13—C14—F1	179.4 (3)	C40—C41—C42—F4	176.8 (2)
C12—C13—C14—C15	−0.1 (5)	C40—C41—C42—C43	−2.8 (4)
F1—C14—C15—C16	179.8 (2)	F4—C42—C43—C44	−177.7 (2)
C13—C14—C15—C16	−0.7 (4)	C41—C42—C43—C44	1.9 (4)
C12—C11—C16—C15	0.9 (4)	C40—C39—C44—C43	−1.3 (4)
P1—C11—C16—C15	179.29 (19)	P2—C39—C44—C43	174.10 (19)
C14—C15—C16—C11	0.3 (4)	C42—C43—C44—C39	0.2 (4)
C23—P1—C17—C18	164.88 (19)	C51—P2—C45—C46	101.2 (2)
C11—P1—C17—C18	−86.5 (2)	C39—P2—C45—C46	−4.1 (2)
Mo1—P1—C17—C18	39.3 (2)	Mo2—P2—C45—C46	−131.89 (18)
C23—P1—C17—C22	−19.6 (2)	C51—P2—C45—C50	−78.1 (2)
C11—P1—C17—C22	89.0 (2)	C39—P2—C45—C50	176.65 (19)
Mo1—P1—C17—C22	−145.17 (17)	Mo2—P2—C45—C50	48.9 (2)
C22—C17—C18—C19	−1.2 (4)	C50—C45—C46—C47	−0.7 (3)
P1—C17—C18—C19	174.5 (2)	P2—C45—C46—C47	−179.96 (18)
C17—C18—C19—C20	2.4 (4)	C45—C46—C47—C48	−0.2 (4)
C18—C19—C20—F2	178.9 (2)	C46—C47—C48—F5	−177.3 (2)
C18—C19—C20—C21	−1.1 (4)	C46—C47—C48—C49	1.2 (4)
F2—C20—C21—C22	178.5 (2)	F5—C48—C49—C50	177.4 (2)
C19—C20—C21—C22	−1.5 (4)	C47—C48—C49—C50	−1.2 (4)
C20—C21—C22—C17	2.8 (4)	C48—C49—C50—C45	0.1 (4)
C18—C17—C22—C21	−1.5 (4)	C46—C45—C50—C49	0.8 (4)
P1—C17—C22—C21	−176.99 (19)	P2—C45—C50—C49	−179.95 (19)
C17—P1—C23—C28	121.0 (2)	C45—P2—C51—C52	−10.9 (2)
C11—P1—C23—C28	14.8 (2)	C39—P2—C51—C52	96.2 (2)
Mo1—P1—C23—C28	−110.42 (19)	Mo2—P2—C51—C52	−135.61 (17)
C17—P1—C23—C24	−59.7 (2)	C45—P2—C51—C56	171.36 (19)
C11—P1—C23—C24	−165.84 (18)	C39—P2—C51—C56	−81.6 (2)
Mo1—P1—C23—C24	68.90 (19)	Mo2—P2—C51—C56	46.6 (2)
C28—C23—C24—C25	−0.9 (4)	C56—C51—C52—C53	−0.5 (3)
P1—C23—C24—C25	179.73 (19)	P2—C51—C52—C53	−178.30 (18)
C23—C24—C25—C26	2.5 (4)	C51—C52—C53—C54	0.4 (4)
C24—C25—C26—F3	177.5 (2)	C52—C53—C54—F6	179.7 (2)
C24—C25—C26—C27	−2.2 (4)	C52—C53—C54—C55	0.2 (4)
C25—C26—C27—C28	0.4 (4)	F6—C54—C55—C56	179.9 (2)
F3—C26—C27—C28	−179.3 (2)	C53—C54—C55—C56	−0.6 (4)
C24—C23—C28—C27	−1.0 (3)	C54—C55—C56—C51	0.5 (4)
P1—C23—C28—C27	178.31 (18)	C52—C51—C56—C55	0.1 (3)
C26—C27—C28—C23	1.3 (4)	P2—C51—C56—C55	177.88 (19)

### Dicarbonyl(η5-cyclopentadienyl)propionyl[tris(4-fluorophenyl)phosphane-κP]molybdenum(II) (2) Hydrogen-bond geometry (Å, º)

**Table:**

*D*—H···*A*	*D*—H	H···*A*	*D*···*A*	*D*—H···*A*
C15—H15···O6	0.95	2.59	3.371 (4)	139
C49—H49···F4^i^	0.95	2.55	3.344 (3)	141
C55—H55···O3^ii^	0.95	2.53	3.450 (3)	164
C34—H34···O1^iii^	1.00	2.38	3.237 (3)	143

Symmetry codes: (i) *x*+1, *y*, *z*; (ii) *x*−1, *y*, *z*; (iii) *x*, −*y*+1/2, *z*+1/2.

### Dicarbonyl(η5-cyclopentadienyl)propionyl[tris(4-methoxyphenyl)phosphane-κP]molybdenum(II) dichloromethane solvate (3) Crystal data

**Table:**

[Mo(C_5_H_5_)(C_3_H_5_O)(C_21_H_21_O_3_P)(CO)_2_]·CH_2_Cl_2_	*Z* = 2
*M**_r_* = 711.39	*F*(000) = 728
Triclinic, *P*1	*D*_x_ = 1.508 Mg m^−^^3^
*a* = 10.5308 (6) Å	Mo *K*α radiation, λ = 0.71073 Å
*b* = 12.1305 (7) Å	Cell parameters from 9554 reflections
*c* = 13.6154 (8) Å	θ = 3.0–36.3°
α = 97.660 (2)°	µ = 0.68 mm^−^^1^
β = 104.759 (2)°	*T* = 170 K
γ = 107.081 (2)°	Block, pale yellow
*V* = 1566.43 (16) Å^3^	0.23 × 0.21 × 0.12 mm

### Dicarbonyl(η5-cyclopentadienyl)propionyl[tris(4-methoxyphenyl)phosphane-κP]molybdenum(II) dichloromethane solvate (3) Data collection

**Table:**

Bruker D8 QUEST ECO diffractometer	9578 independent reflections
Radiation source: sealed tube, Siemens KFFMO2K-90C	9053 reflections with *I* > 2σ(*I*)
Curved Graphite monochromator	*R*_int_ = 0.029
Detector resolution: 7.3910 pixels mm^-1^	θ_max_ = 30.5°, θ_min_ = 2.3°
φ and ω scans	*h* = −15→15
Absorption correction: multi-scan (Krause *et al.*, 2015)	*k* = −17→17
*T*_min_ = 0.84, *T*_max_ = 0.92	*l* = −19→19
81820 measured reflections	

### Dicarbonyl(η5-cyclopentadienyl)propionyl[tris(4-methoxyphenyl)phosphane-κP]molybdenum(II) dichloromethane solvate (3) Refinement

**Table:**

Refinement on *F*^2^	Primary atom site location: dual
Least-squares matrix: full	Secondary atom site location: difference Fourier map
*R*[*F*^2^ > 2σ(*F*^2^)] = 0.020	Hydrogen site location: inferred from neighbouring sites
*wR*(*F*^2^) = 0.054	H-atom parameters constrained
*S* = 1.06	*w* = 1/[σ^2^(*F*_o_^2^) + (0.0226*P*)^2^ + 0.7135*P*] where *P* = (*F*_o_^2^ + 2*F*_c_^2^)/3
9578 reflections	(Δ/σ)_max_ = 0.001
393 parameters	Δρ_max_ = 0.40 e Å^−^^3^
0 restraints	Δρ_min_ = −0.48 e Å^−^^3^

### Dicarbonyl(η5-cyclopentadienyl)propionyl[tris(4-methoxyphenyl)phosphane-κP]molybdenum(II) dichloromethane solvate (3) Special details

**Table:**

Geometry. All esds (except the esd in the dihedral angle between two l.s. planes) are estimated using the full covariance matrix. The cell esds are taken into account individually in the estimation of esds in distances, angles and torsion angles; correlations between esds in cell parameters are only used when they are defined by crystal symmetry. An approximate (isotropic) treatment of cell esds is used for estimating esds involving l.s. planes.

### Dicarbonyl(η5-cyclopentadienyl)propionyl[tris(4-methoxyphenyl)phosphane-κP]molybdenum(II) dichloromethane solvate (3) Fractional atomic coordinates and isotropic or equivalent isotropic displacement parameters (Å^2^)

**Table:**

	*x*	*y*	*z*	*U*_iso_*/*U*_eq_	Occ. (<1)
Mo1	0.52042 (2)	0.25393 (2)	0.09427 (2)	0.01619 (3)	
P1	0.51816 (3)	0.35557 (2)	0.26360 (2)	0.01580 (5)	
O1	0.68635 (12)	0.51311 (8)	0.09342 (8)	0.0368 (2)	
O2	0.71687 (11)	0.15626 (9)	0.24292 (8)	0.0355 (2)	
O3	0.65766 (10)	0.19845 (9)	−0.07044 (7)	0.0330 (2)	
O4	0.07178 (10)	0.57231 (9)	0.24125 (8)	0.0335 (2)	
O5	0.41490 (10)	0.06671 (8)	0.57748 (7)	0.03008 (19)	
O6	1.00779 (9)	0.78117 (8)	0.52048 (7)	0.03004 (19)	
C1	0.62823 (12)	0.41791 (10)	0.09761 (9)	0.0227 (2)	
C2	0.64709 (12)	0.19679 (10)	0.19028 (8)	0.0220 (2)	
C3	0.68821 (12)	0.24759 (10)	0.02074 (9)	0.0233 (2)	
C4	0.84445 (13)	0.30859 (12)	0.07936 (10)	0.0301 (2)	
H4A	0.878597	0.383496	0.057367	0.036*	
H4B	0.857453	0.328655	0.154986	0.036*	
C5	0.93197 (15)	0.23248 (14)	0.06042 (13)	0.0385 (3)	
H5A	0.909437	0.164404	0.092354	0.058*	
H5B	1.031399	0.27963	0.091479	0.058*	
H5C	0.911323	0.204489	−0.01474	0.058*	
C6	0.32457 (13)	0.07480 (10)	0.03752 (10)	0.0266 (2)	
H6	0.313597	0.0047	0.069965	0.032*	
C7	0.27424 (12)	0.16854 (10)	0.05911 (9)	0.0246 (2)	
H7	0.222345	0.176265	0.110302	0.03*	
C8	0.29947 (13)	0.24425 (11)	−0.00983 (10)	0.0272 (2)	
H8	0.266202	0.312821	−0.017144	0.033*	
C9	0.36793 (14)	0.19845 (12)	−0.07342 (9)	0.0309 (3)	
H9	0.388632	0.227539	−0.13493	0.037*	
C10	0.38374 (14)	0.09369 (11)	−0.04378 (10)	0.0306 (3)	
H10	0.418501	0.037449	−0.080607	0.037*	
C11	0.37443 (11)	0.41395 (9)	0.25087 (8)	0.01780 (18)	
C12	0.35998 (12)	0.49063 (10)	0.18422 (9)	0.0226 (2)	
H12	0.419737	0.505677	0.142103	0.027*	
C13	0.26014 (12)	0.54555 (11)	0.17798 (9)	0.0237 (2)	
H13	0.252561	0.598157	0.132747	0.028*	
C14	0.17141 (12)	0.52242 (10)	0.23893 (9)	0.0227 (2)	
C15	0.18237 (12)	0.44423 (11)	0.30402 (9)	0.0248 (2)	
H15	0.120263	0.426933	0.344156	0.03*	
C16	0.28358 (12)	0.39144 (10)	0.31053 (9)	0.02147 (19)	
H16	0.291252	0.339271	0.356177	0.026*	
C17	0.05341 (16)	0.65233 (14)	0.17571 (14)	0.0397 (3)	
H17A	0.141582	0.718094	0.192016	0.06*	
H17B	−0.018996	0.683173	0.187415	0.06*	
H17C	0.025013	0.610782	0.102594	0.06*	
C18	0.49824 (11)	0.26789 (9)	0.36153 (8)	0.01745 (17)	
C19	0.40076 (12)	0.15263 (10)	0.33211 (9)	0.0235 (2)	
H19	0.351772	0.118227	0.26037	0.028*	
C20	0.37468 (13)	0.08818 (10)	0.40562 (9)	0.0253 (2)	
H20	0.306101	0.011206	0.384356	0.03*	
C21	0.44904 (12)	0.13610 (10)	0.51117 (8)	0.02071 (19)	
C22	0.54956 (12)	0.24834 (10)	0.54166 (8)	0.02109 (19)	
H22	0.602748	0.280472	0.612987	0.025*	
C23	0.57210 (11)	0.31379 (10)	0.46696 (8)	0.02006 (19)	
H23	0.639338	0.391388	0.488536	0.024*	
C24	0.48069 (19)	0.11574 (13)	0.68676 (10)	0.0395 (3)	
H24A	0.443749	0.058545	0.725802	0.059*	
H24B	0.461623	0.188617	0.7061	0.059*	
H24C	0.581792	0.133654	0.703274	0.059*	
C25	0.66885 (11)	0.48601 (9)	0.33776 (8)	0.01716 (17)	
C26	0.65268 (12)	0.58564 (10)	0.39084 (9)	0.0221 (2)	
H26	0.561347	0.586707	0.38549	0.027*	
C27	0.76725 (12)	0.68248 (10)	0.45087 (9)	0.0241 (2)	
H27	0.75409	0.749483	0.485784	0.029*	
C28	0.90179 (12)	0.68197 (10)	0.46023 (8)	0.02111 (19)	
C29	0.92037 (12)	0.58325 (11)	0.40946 (9)	0.0246 (2)	
H29	1.011851	0.581542	0.416595	0.03*	
C30	0.80430 (12)	0.48711 (10)	0.34821 (9)	0.0231 (2)	
H30	0.817702	0.420618	0.312657	0.028*	
C31	1.14412 (14)	0.79549 (14)	0.51248 (11)	0.0366 (3)	
H31A	1.178982	0.737871	0.54421	0.055*	
H31B	1.207379	0.875805	0.548891	0.055*	
H31C	1.139505	0.782624	0.438867	0.055*	
C32	−0.0396 (2)	0.01902 (17)	0.26603 (16)	0.0533 (4)	
H32A	0.017643	0.101022	0.305243	0.064*	0.532 (15)
H32B	−0.133229	0.018994	0.22801	0.064*	0.532 (15)
H32C	−0.117475	0.043473	0.229775	0.064*	0.468 (15)
H32D	0.030037	0.088435	0.319629	0.064*	0.468 (15)
Cl1A	−0.0560 (9)	−0.0707 (4)	0.3518 (4)	0.0661 (11)	0.532 (15)
Cl1B	−0.1046 (5)	−0.0962 (5)	0.3280 (3)	0.0714 (6)	0.468 (15)
Cl2	0.03837 (4)	−0.02605 (4)	0.17571 (4)	0.05013 (10)	

### Dicarbonyl(η5-cyclopentadienyl)propionyl[tris(4-methoxyphenyl)phosphane-κP]molybdenum(II) dichloromethane solvate (3) Atomic displacement parameters (Å^2^)

**Table:**

	*U*^11^	*U*^22^	*U*^33^	*U*^12^	*U*^13^	*U*^23^
Mo1	0.01913 (4)	0.01478 (4)	0.01330 (4)	0.00475 (3)	0.00476 (3)	0.00160 (3)
P1	0.01702 (11)	0.01515 (11)	0.01494 (11)	0.00486 (9)	0.00573 (9)	0.00235 (8)
O1	0.0471 (6)	0.0231 (4)	0.0411 (5)	0.0065 (4)	0.0188 (5)	0.0125 (4)
O2	0.0443 (6)	0.0374 (5)	0.0274 (4)	0.0239 (5)	0.0040 (4)	0.0076 (4)
O3	0.0351 (5)	0.0415 (5)	0.0210 (4)	0.0113 (4)	0.0121 (4)	−0.0003 (4)
O4	0.0338 (5)	0.0424 (5)	0.0407 (5)	0.0255 (4)	0.0204 (4)	0.0176 (4)
O5	0.0403 (5)	0.0263 (4)	0.0217 (4)	0.0052 (4)	0.0110 (4)	0.0102 (3)
O6	0.0227 (4)	0.0239 (4)	0.0318 (5)	−0.0006 (3)	0.0043 (3)	−0.0042 (3)
C1	0.0274 (5)	0.0218 (5)	0.0199 (5)	0.0083 (4)	0.0093 (4)	0.0050 (4)
C2	0.0271 (5)	0.0193 (5)	0.0183 (4)	0.0080 (4)	0.0068 (4)	0.0005 (4)
C3	0.0272 (5)	0.0224 (5)	0.0216 (5)	0.0083 (4)	0.0104 (4)	0.0039 (4)
C4	0.0255 (6)	0.0321 (6)	0.0292 (6)	0.0057 (5)	0.0119 (5)	−0.0018 (5)
C5	0.0319 (7)	0.0378 (7)	0.0445 (8)	0.0145 (6)	0.0096 (6)	0.0034 (6)
C6	0.0246 (5)	0.0185 (5)	0.0283 (6)	0.0014 (4)	0.0026 (4)	0.0013 (4)
C7	0.0198 (5)	0.0247 (5)	0.0238 (5)	0.0035 (4)	0.0037 (4)	0.0029 (4)
C8	0.0242 (5)	0.0274 (6)	0.0249 (5)	0.0074 (4)	0.0003 (4)	0.0068 (4)
C9	0.0310 (6)	0.0369 (7)	0.0157 (5)	0.0044 (5)	0.0017 (4)	0.0027 (4)
C10	0.0311 (6)	0.0263 (6)	0.0237 (5)	0.0042 (5)	0.0037 (5)	−0.0077 (4)
C11	0.0175 (4)	0.0184 (4)	0.0171 (4)	0.0056 (4)	0.0060 (3)	0.0027 (3)
C12	0.0228 (5)	0.0263 (5)	0.0234 (5)	0.0101 (4)	0.0114 (4)	0.0094 (4)
C13	0.0248 (5)	0.0258 (5)	0.0250 (5)	0.0113 (4)	0.0102 (4)	0.0097 (4)
C14	0.0203 (5)	0.0247 (5)	0.0244 (5)	0.0098 (4)	0.0076 (4)	0.0038 (4)
C15	0.0238 (5)	0.0309 (6)	0.0254 (5)	0.0115 (4)	0.0133 (4)	0.0091 (4)
C16	0.0219 (5)	0.0245 (5)	0.0206 (5)	0.0084 (4)	0.0093 (4)	0.0075 (4)
C17	0.0368 (7)	0.0399 (7)	0.0557 (9)	0.0245 (6)	0.0182 (7)	0.0213 (7)
C18	0.0190 (4)	0.0173 (4)	0.0168 (4)	0.0065 (4)	0.0064 (3)	0.0038 (3)
C19	0.0270 (5)	0.0197 (5)	0.0178 (5)	0.0029 (4)	0.0035 (4)	0.0027 (4)
C20	0.0289 (6)	0.0189 (5)	0.0222 (5)	0.0017 (4)	0.0056 (4)	0.0044 (4)
C21	0.0249 (5)	0.0210 (5)	0.0202 (5)	0.0098 (4)	0.0099 (4)	0.0073 (4)
C22	0.0241 (5)	0.0226 (5)	0.0157 (4)	0.0074 (4)	0.0059 (4)	0.0035 (4)
C23	0.0213 (5)	0.0188 (4)	0.0181 (4)	0.0046 (4)	0.0062 (4)	0.0029 (4)
C24	0.0615 (10)	0.0355 (7)	0.0211 (6)	0.0107 (7)	0.0169 (6)	0.0103 (5)
C25	0.0182 (4)	0.0158 (4)	0.0169 (4)	0.0044 (3)	0.0069 (3)	0.0020 (3)
C26	0.0199 (5)	0.0195 (5)	0.0263 (5)	0.0069 (4)	0.0085 (4)	−0.0001 (4)
C27	0.0245 (5)	0.0179 (5)	0.0276 (5)	0.0066 (4)	0.0082 (4)	−0.0013 (4)
C28	0.0214 (5)	0.0192 (5)	0.0187 (4)	0.0026 (4)	0.0057 (4)	0.0020 (4)
C29	0.0182 (5)	0.0273 (5)	0.0252 (5)	0.0062 (4)	0.0068 (4)	−0.0010 (4)
C30	0.0206 (5)	0.0224 (5)	0.0244 (5)	0.0077 (4)	0.0073 (4)	−0.0028 (4)
C31	0.0217 (6)	0.0409 (7)	0.0324 (6)	−0.0034 (5)	0.0045 (5)	−0.0004 (5)
C32	0.0701 (12)	0.0490 (10)	0.0572 (11)	0.0328 (9)	0.0283 (9)	0.0210 (8)
Cl1A	0.104 (3)	0.0631 (13)	0.0563 (13)	0.0427 (15)	0.0394 (16)	0.0343 (11)
Cl1B	0.0648 (14)	0.0990 (17)	0.0501 (10)	0.0193 (13)	0.0146 (10)	0.0415 (9)
Cl2	0.03877 (19)	0.0537 (2)	0.0484 (2)	0.00556 (17)	0.01330 (16)	0.00379 (18)

### Dicarbonyl(η5-cyclopentadienyl)propionyl[tris(4-methoxyphenyl)phosphane-κP]molybdenum(II) dichloromethane solvate (3) Geometric parameters (Å, º)

**Table:**

Mo1—P1	2.4745 (3)	C13—C14	1.3942 (16)
Mo1—C1	1.9675 (12)	C13—H13	0.95
Mo1—C2	1.9658 (12)	C14—C15	1.3908 (16)
Mo1—C3	2.2564 (11)	C15—C16	1.3863 (16)
Mo1—C9	2.3092 (12)	C15—H15	0.95
Mo1—C10	2.3210 (12)	C16—H16	0.95
Mo1—C8	2.3562 (12)	C17—H17A	0.98
Mo1—C6	2.3833 (12)	C17—H17B	0.98
Mo1—C7	2.3835 (11)	C17—H17C	0.98
P1—C25	1.8232 (11)	C18—C23	1.3925 (14)
P1—C18	1.8283 (11)	C18—C19	1.4026 (15)
P1—C11	1.8307 (11)	C19—C20	1.3823 (16)
O1—C1	1.1540 (15)	C19—H19	0.95
O2—C2	1.1547 (15)	C20—C21	1.3974 (16)
O3—C3	1.2192 (14)	C20—H20	0.95
O4—C14	1.3612 (14)	C21—C22	1.3876 (15)
O4—C17	1.4246 (17)	C22—C23	1.3959 (15)
O5—C21	1.3576 (13)	C22—H22	0.95
O5—C24	1.4298 (16)	C23—H23	0.95
O6—C28	1.3595 (13)	C24—H24A	0.98
O6—C31	1.4287 (16)	C24—H24B	0.98
C3—C4	1.5329 (17)	C24—H24C	0.98
C4—C5	1.5251 (19)	C25—C30	1.3928 (15)
C4—H4A	0.99	C25—C26	1.4009 (14)
C4—H4B	0.99	C26—C27	1.3830 (15)
C5—H5A	0.98	C26—H26	0.95
C5—H5B	0.98	C27—C28	1.3917 (16)
C5—H5C	0.98	C27—H27	0.95
C6—C10	1.4146 (18)	C28—C29	1.3914 (16)
C6—C7	1.4159 (17)	C29—C30	1.3905 (16)
C6—H6	1.0	C29—H29	0.95
C7—C8	1.4172 (17)	C30—H30	0.95
C7—H7	1.0	C31—H31A	0.98
C8—C9	1.4153 (19)	C31—H31B	0.98
C8—H8	1.0	C31—H31C	0.98
C9—C10	1.426 (2)	C32—Cl1A	1.705 (3)
C9—H9	1.0	C32—Cl2	1.7561 (19)
C10—H10	1.0	C32—Cl1B	1.783 (4)
C11—C12	1.3963 (15)	C32—H32A	0.99
C11—C16	1.3970 (14)	C32—H32B	0.99
C12—C13	1.3920 (15)	C32—H32C	0.99
C12—H12	0.95	C32—H32D	0.99
			
C1—Mo1—C2	106.36 (5)	C9—C10—Mo1	71.62 (7)
C1—Mo1—C3	72.49 (4)	C6—C10—H10	125.7
C2—Mo1—C3	74.79 (4)	C9—C10—H10	125.7
C2—Mo1—C9	140.57 (5)	Mo1—C10—H10	125.7
C1—Mo1—C9	100.33 (5)	C12—C11—C16	118.13 (10)
C3—Mo1—C9	86.50 (5)	C12—C11—P1	119.07 (8)
C2—Mo1—C10	106.27 (5)	C16—C11—P1	122.64 (8)
C1—Mo1—C10	131.51 (5)	C13—C12—C11	121.61 (10)
C3—Mo1—C10	82.71 (5)	C13—C12—H12	119.2
C9—Mo1—C10	35.86 (5)	C11—C12—H12	119.2
C2—Mo1—C8	153.82 (5)	C12—C13—C14	119.18 (10)
C1—Mo1—C8	99.03 (5)	C12—C13—H13	120.4
C3—Mo1—C8	120.03 (4)	C14—C13—H13	120.4
C9—Mo1—C8	35.30 (5)	O4—C14—C15	115.39 (10)
C10—Mo1—C8	58.80 (5)	O4—C14—C13	124.68 (11)
C2—Mo1—C6	96.95 (5)	C15—C14—C13	119.93 (10)
C1—Mo1—C6	156.61 (5)	C16—C15—C14	120.25 (10)
C3—Mo1—C6	112.97 (4)	C16—C15—H15	119.9
C9—Mo1—C6	58.69 (5)	C14—C15—H15	119.9
C10—Mo1—C6	34.97 (5)	C15—C16—C11	120.87 (10)
C8—Mo1—C6	58.10 (4)	C15—C16—H16	119.6
C2—Mo1—C7	119.76 (5)	C11—C16—H16	119.6
C1—Mo1—C7	127.63 (5)	O4—C17—H17A	109.5
C3—Mo1—C7	140.20 (4)	O4—C17—H17B	109.5
C9—Mo1—C7	58.32 (4)	H17A—C17—H17B	109.5
C10—Mo1—C7	58.06 (4)	O4—C17—H17C	109.5
C8—Mo1—C7	34.79 (4)	H17A—C17—H17C	109.5
C6—Mo1—C7	34.56 (4)	H17B—C17—H17C	109.5
C1—Mo1—P1	79.97 (3)	C23—C18—C19	117.86 (10)
C2—Mo1—P1	79.79 (3)	C23—C18—P1	122.10 (8)
C3—Mo1—P1	134.90 (3)	C19—C18—P1	119.96 (8)
C9—Mo1—P1	133.97 (4)	C20—C19—C18	121.19 (10)
C10—Mo1—P1	140.75 (3)	C20—C19—H19	119.4
C8—Mo1—P1	98.76 (3)	C18—C19—H19	119.4
C6—Mo1—P1	106.54 (3)	C19—C20—C21	120.10 (10)
C7—Mo1—P1	84.85 (3)	C19—C20—H20	120.0
C25—P1—C18	102.11 (5)	C21—C20—H20	120.0
C25—P1—C11	101.79 (5)	O5—C21—C22	124.58 (10)
C18—P1—C11	102.80 (5)	O5—C21—C20	115.76 (10)
C25—P1—Mo1	117.73 (3)	C22—C21—C20	119.66 (10)
C18—P1—Mo1	117.30 (3)	C21—C22—C23	119.65 (10)
C11—P1—Mo1	112.88 (3)	C21—C22—H22	120.2
C14—O4—C17	118.55 (10)	C23—C22—H22	120.2
C21—O5—C24	117.63 (10)	C18—C23—C22	121.48 (10)
C28—O6—C31	117.18 (10)	C18—C23—H23	119.3
O1—C1—Mo1	175.82 (11)	C22—C23—H23	119.3
O2—C2—Mo1	175.76 (10)	O5—C24—H24A	109.5
O3—C3—C4	116.42 (11)	O5—C24—H24B	109.5
O3—C3—Mo1	120.75 (9)	H24A—C24—H24B	109.5
C4—C3—Mo1	122.76 (8)	O5—C24—H24C	109.5
C5—C4—C3	113.12 (11)	H24A—C24—H24C	109.5
C5—C4—H4A	109.0	H24B—C24—H24C	109.5
C3—C4—H4A	109.0	C30—C25—C26	117.80 (10)
C5—C4—H4B	109.0	C30—C25—P1	120.87 (8)
C3—C4—H4B	109.0	C26—C25—P1	121.23 (8)
H4A—C4—H4B	107.8	C27—C26—C25	121.21 (10)
C4—C5—H5A	109.5	C27—C26—H26	119.4
C4—C5—H5B	109.5	C25—C26—H26	119.4
H5A—C5—H5B	109.5	C26—C27—C28	120.13 (10)
C4—C5—H5C	109.5	C26—C27—H27	119.9
H5A—C5—H5C	109.5	C28—C27—H27	119.9
H5B—C5—H5C	109.5	O6—C28—C29	124.40 (10)
C10—C6—C7	107.57 (11)	O6—C28—C27	115.94 (10)
C10—C6—Mo1	70.11 (7)	C29—C28—C27	119.66 (10)
C7—C6—Mo1	72.73 (7)	C30—C29—C28	119.63 (10)
C10—C6—H6	126.1	C30—C29—H29	120.2
C7—C6—H6	126.1	C28—C29—H29	120.2
Mo1—C6—H6	126.1	C29—C30—C25	121.55 (10)
C6—C7—C8	108.64 (11)	C29—C30—H30	119.2
C6—C7—Mo1	72.71 (7)	C25—C30—H30	119.2
C8—C7—Mo1	71.55 (7)	O6—C31—H31A	109.5
C6—C7—H7	125.6	O6—C31—H31B	109.5
C8—C7—H7	125.6	H31A—C31—H31B	109.5
Mo1—C7—H7	125.6	O6—C31—H31C	109.5
C9—C8—C7	107.71 (11)	H31A—C31—H31C	109.5
C9—C8—Mo1	70.54 (7)	H31B—C31—H31C	109.5
C7—C8—Mo1	73.66 (7)	Cl1A—C32—Cl2	112.38 (13)
C9—C8—H8	126.0	Cl2—C32—Cl1B	111.88 (16)
C7—C8—H8	126.0	Cl1A—C32—H32A	109.1
Mo1—C8—H8	126.0	Cl2—C32—H32A	109.1
C8—C9—C10	107.85 (11)	Cl1A—C32—H32B	109.1
C8—C9—Mo1	74.16 (7)	Cl2—C32—H32B	109.1
C10—C9—Mo1	72.52 (7)	H32A—C32—H32B	107.9
C8—C9—H9	125.8	Cl2—C32—H32C	109.2
C10—C9—H9	125.8	Cl1B—C32—H32C	109.2
Mo1—C9—H9	125.8	Cl2—C32—H32D	109.2
C6—C10—C9	108.20 (11)	Cl1B—C32—H32D	109.2
C6—C10—Mo1	74.93 (7)	H32C—C32—H32D	107.9
			
O3—C3—C4—C5	−45.68 (17)	C11—P1—C18—C23	−94.92 (9)
Mo1—C3—C4—C5	137.17 (10)	Mo1—P1—C18—C23	140.61 (8)
C10—C6—C7—C8	1.13 (13)	C25—P1—C18—C19	−173.09 (9)
Mo1—C6—C7—C8	62.95 (8)	C11—P1—C18—C19	81.66 (10)
C10—C6—C7—Mo1	−61.82 (8)	Mo1—P1—C18—C19	−42.82 (10)
C6—C7—C8—C9	−0.97 (13)	C23—C18—C19—C20	2.47 (17)
Mo1—C7—C8—C9	62.73 (8)	P1—C18—C19—C20	−174.25 (10)
C6—C7—C8—Mo1	−63.70 (8)	C18—C19—C20—C21	−2.02 (19)
C7—C8—C9—C10	0.43 (14)	C24—O5—C21—C22	4.22 (18)
Mo1—C8—C9—C10	65.20 (9)	C24—O5—C21—C20	−175.32 (12)
C7—C8—C9—Mo1	−64.78 (8)	C19—C20—C21—O5	179.25 (11)
C7—C6—C10—C9	−0.86 (14)	C19—C20—C21—C22	−0.32 (18)
Mo1—C6—C10—C9	−64.38 (8)	O5—C21—C22—C23	−177.41 (11)
C7—C6—C10—Mo1	63.52 (8)	C20—C21—C22—C23	2.11 (17)
C8—C9—C10—C6	0.27 (14)	C19—C18—C23—C22	−0.64 (16)
Mo1—C9—C10—C6	66.56 (9)	P1—C18—C23—C22	176.00 (8)
C8—C9—C10—Mo1	−66.29 (9)	C21—C22—C23—C18	−1.63 (17)
C25—P1—C11—C12	71.91 (9)	C18—P1—C25—C30	86.31 (10)
C18—P1—C11—C12	177.41 (9)	C11—P1—C25—C30	−167.65 (9)
Mo1—P1—C11—C12	−55.25 (9)	Mo1—P1—C25—C30	−43.70 (10)
C25—P1—C11—C16	−103.61 (10)	C18—P1—C25—C26	−90.06 (10)
C18—P1—C11—C16	1.89 (10)	C11—P1—C25—C26	15.98 (10)
Mo1—P1—C11—C16	129.22 (8)	Mo1—P1—C25—C26	139.94 (8)
C16—C11—C12—C13	1.21 (17)	C30—C25—C26—C27	0.61 (17)
P1—C11—C12—C13	−174.52 (9)	P1—C25—C26—C27	177.08 (9)
C11—C12—C13—C14	−0.71 (18)	C25—C26—C27—C28	−0.54 (18)
C17—O4—C14—C15	−179.30 (12)	C31—O6—C28—C29	14.43 (17)
C17—O4—C14—C13	1.31 (19)	C31—O6—C28—C27	−165.75 (12)
C12—C13—C14—O4	178.64 (11)	C26—C27—C28—O6	179.75 (11)
C12—C13—C14—C15	−0.72 (18)	C26—C27—C28—C29	−0.42 (18)
O4—C14—C15—C16	−177.78 (11)	O6—C28—C29—C30	−178.90 (11)
C13—C14—C15—C16	1.64 (18)	C27—C28—C29—C30	1.28 (18)
C14—C15—C16—C11	−1.13 (18)	C28—C29—C30—C25	−1.22 (19)
C12—C11—C16—C15	−0.28 (17)	C26—C25—C30—C29	0.27 (17)
P1—C11—C16—C15	175.28 (9)	P1—C25—C30—C29	−176.21 (9)
C25—P1—C18—C23	10.34 (10)		

### Dicarbonyl(η5-cyclopentadienyl)propionyl[tris(4-methoxyphenyl)phosphane-κP]molybdenum(II) dichloromethane solvate (3) Hydrogen-bond geometry (Å, º)

**Table:**

*D*—H···*A*	*D*—H	H···*A*	*D*···*A*	*D*—H···*A*
C31—H31*B*···O5^i^	0.98	2.58	3.4880 (16)	155
C6—H6···O3^ii^	1.00	2.57	3.4555 (16)	148
C8—H8···O1^iii^	1.00	2.45	3.2714 (16)	139
C32—H32*B*···O2^iv^	0.99	2.63	3.418 (2)	137

Symmetry codes: (i) *x*+1, *y*+1, *z*; (ii) −*x*+1, −*y*, −*z*; (iii) −*x*+1, −*y*+1, −*z*; (iv) *x*−1, *y*, *z*.
